# Silent forces, hidden currents: the influence of static magnetic field stimulation on tumor biophysics

**DOI:** 10.1038/s44385-026-00071-z

**Published:** 2026-03-06

**Authors:** Prerna Verma, Amogh Varshney, Mahwish Lais, Aarat P. Kalra

**Affiliations:** 1 Centre for Biomedical Engineering, Indian Institute of Technology Delhi, New Delhi, India; 2 Department of Biomedical Engineering, All India Institute of Medical Sciences Delhi, New Delhi, India; 3 Amar Nath and Shashi Khosla School of Information Technology, Indian Institute of Technology Delhi, New Delhi, India

**Keywords:** Biophysics, Nanoparticles, Translational research

## Abstract

One of the prime goals of medical oncology is to find cost-effective, non-invasive cancer treatment modalities with negligible side effects. Electroceutical modalities promise to fulfill these criteria, but a precise understanding of their molecular targets is mostly unclear, limiting efficacy. Here, we review static magnetic field (SMF) stimulation for tumor treatment as a possible electroceutical modality. We start by discussing the biophysical principles through which SMFs interact with known biochemical targets and discuss evidence in support of their use for tumor treatment. We analyze the different modes of SMF stimulation and demonstrate that apparent contradictions in reported mechanisms and therapeutic outcomes are primarily due to differences in physiological conditions and biophysical stimulation parameters across studies. Finally, we provide a list of open questions that must be addressed for the success of SMF stimulation as an electroceutical modality.

## Introduction

Traditional tumor treatment modalities such as surgery, chemotherapy and radiation therapy have been successful at decreasing cancer-related mortality^1^, but are associated with numerous side effects (including infertility, nausea, fatigue, and even an increased risk of secondary malignancy)^2,3^, severely decreasing patient quality of life. Electroceuticals, which involve use of electromagnetic stimuli (encompassing electrical stimuli or magnetic stimuli or a combination of both), representing a promising class of treatment modalities with comparatively limited side effects^4–7^. Indeed, tumor-treating electric field (TTField) therapy for cancer treatment^8^, transcutaneous electrical nerve stimulation for relieving nociceptive, neuropathic, and musculoskeletal pain^9,10^, and pulsed electromagnetic field therapy for bone healing^11^ have attained FDA approval in various capacities for mass utilization, and are associated with mild or negligible side effects^4–7^.

Electromagnetic stimuli can be used in a variety of ways to treat tumors. For example, TTField therapy uses 1–2 V/cm, 100–300 kHz AC electric fields to disrupt cell division, which ultimately leads to apoptosis^8^. Reversible electroporation (RE) and irreversible electroporation (IRE) therapies use high-voltage electrical pulses to depolarize the cell membrane and cause its structural damage, allowing the uptake of drugs such as cisplatin (in RE therapy) or cause loss of homeostasis, which eventually leads to cell death (in IRE therapy)^12,13^. Photodynamic and photothermal therapies induce redox and thermal stress which ultimately lead to cell death^14,15^. Although the mechanisms and biochemical signaling cascades targeted by electrical component of electromagnetic stimuli in tumors are well researched, those targeted by magnetic component are not well understood.

**Fig. 1:**
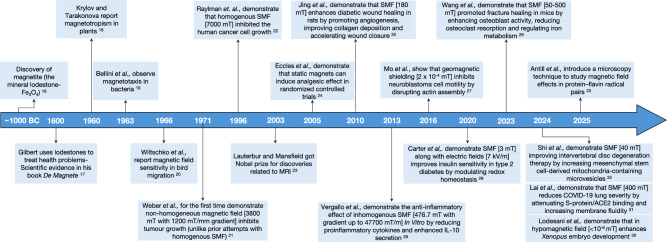
Overview of key milestones in the evolution of SMF research, from fundamental studies to therapeutic investigations in cancer.

Here, we present a biophysical perspective on the present status and opportunities for tumor treatment using static magnetic fields (solely magnetic stimulus). Even though magnetic fields have been known to cause changes in animal physiology for several decades (Fig. 1)^16–33^ and have found extensive use in medicine outside cancer treatment, apparent discrepancies between experiments and conflicting results between studies have made it difficult to understand the modalities in which SMF stimulation could be used for tumor treatment. We collate this data and interpret it through the lens of three mechanisms described in Section “The molecular mechanisms of SMF action". We highlight the cell- and tissue-level signaling cascades that are influenced by SMF stimulation through these mechanisms in Section “The biochemical targets of SMFs”. Notably, we find that SMF stimulation has been reported to enhance the activity of conventional treatments, such as chemotherapy and X-ray-induced radiation therapy, for a variety of biochemical targets. With a view to address the possible side effects of SMF stimulation, we review the consequences of SMF stimulation on noncancer cells, which include altering cell orientation and surface morphology and both increasing and decreasing cell proliferation rates in different cells, in Section “The consequences of SMF stimulation for healthy cells". We also discuss evidence showing that SMF stimulation leads to adverse effects that harm physiology or that it does not influence native physiological systems. Sources behind these discrepancies include inconsistent use and reporting of both physiological and biophysical parameters. when describing results. We discuss these discrepancies and illustrate how they can lead to different results in Section “Unraveling the discrepancies in SMF-induced tumor response”. Finally, we provide our perspective on the optimal regimes of SMF stimulation to use for tumor treatment and highlight open questions that require further investigation at the intersection of SMF stimulation and tumor cell biology in Section “Future outlook and summary".

## The molecular mechanisms of SMF action

**Fig. 2:**
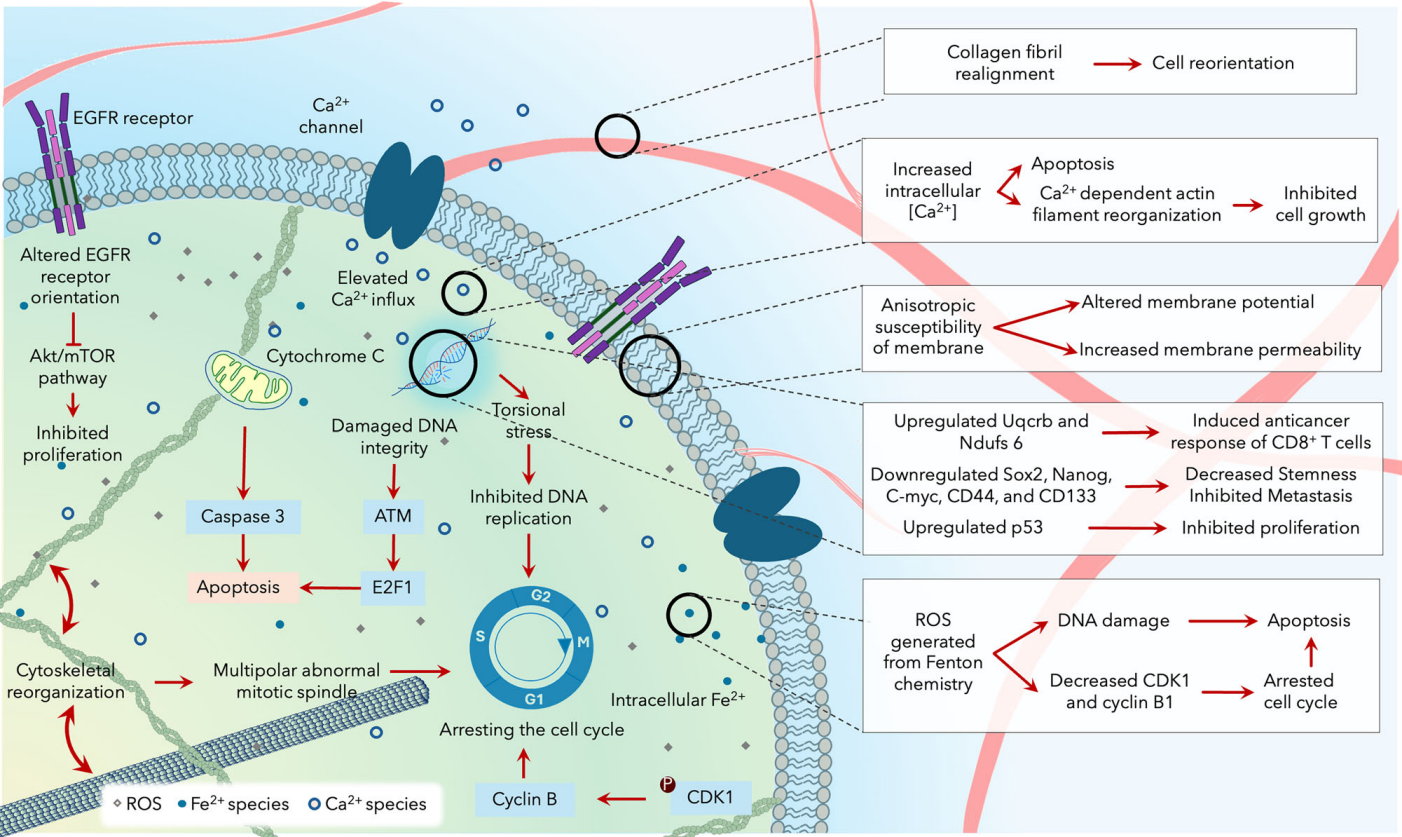
Schematic representation of the various biochemical targets of SMF stimulation.

We find evidence for SMF stimulation influencing biological systems through three biophysical mechanisms, namely, (A) the Lorentz force, (B) magnetic torque, (C) and the radical-pair mechanism (Fig. 2). These biophysical mechanisms lead to changes in the function of a variety of biochemical targets, including collagen fibril alignment, cell membrane potential and integrity, DNA integrity, Ca^2+^ dynamics, cytoskeletal organization, and redox balance.

### The Lorentz force arising from the charge and velocity of a biomolecular assembly

A biomolecular assembly carrying charge *q*, translating with velocity *v*, exposed to a magnetic field *B* and electric field *E* is subjected to a Lorentz Force *F* given by Eq. 1,

1 $$F=q\,\left(E+v\times B\right)$$

When the value of *B* is large enough such that *F* can overcome thermal motion, the Lorentz force can alter trajectories of charged species (including ions, polynucleotides, and proteins) within the cell. Eventually, this disrupts molecular diffusion and, in part, explain the mechanism of action of Transcranial static magnetic stimulation (TMS)^34^. Molecular dynamics simulations suggest that the influence of the Lorentz forces exerted by 1000 mT SMF stimulation on Na^+^ and Cl^−^ ions in aqueous solutions is negligible^35^, so we expect that this force would be applicable to tumor tissue only under the influence of higher SMF intensities.

Experimentally, 16,000 mT SMF stimulation suppressed biomolecular phase-separated condensates of the protein Tau In Vitro through the action of the Lorentz force^36^. 9400 mT SMF stimulation also caused a decrease in DNA synthesis, in part through the Lorentz force in lung adenocarcinoma A459 cell lines^37^.

### The magnetic torque arising from the anisotropic magnetic susceptibility of a biomolecular assembly

The magnetic susceptibility of biomolecular assemblies in the intracellular milieu is typically below zero (*χ*_*m*_ < 0; they are diamagnetic). When exposed to an external magnetic field *B*, change in electron orbital angular momenta ∆*L* in delocalized electron clouds (for example, those present in aromatic residues, peptide bonds and nucleotides) each generate induced magnetic dipole moments ∆*m* in directions opposing the external magnetic field, as shown in Eq. 2.

2 $$\Delta m=-\frac{e}{{2m}_{e}}\Delta L=-\frac{{e}^{2}{r}^{2}}{{4m}_{e}}B$$

3 $$M=\mathop{\mathrm{lim}}\limits_{\Delta V\to 0}\frac{\sum {m}_{i}}{\Delta V}$$

Here, ${m}_{e}$ is the mass of the electron, and *r* is the radius over which it orbits. However, because of the anisotropic distribution of these electron clouds, the resultant induced magnetic dipole moment *M* of the biomolecular assembly (which is a sum of all the individual electron clouds’ magnetic dipole moments) is typically at an angle to the external magnetic field.

The induced magnetic dipole moment itself is also direction dependent (usually represented as a matrix). To minimize its magnetic interaction energy, the biomolecular assembly reorients itself in the presence of an external magnetic field, experiencing a torque *τ* shown by Eq. 4.

4 $$\tau =M\times B$$

For a biomolecular species, the degree of alignment *β* has been expressed as the magnetic interaction energy divided by the thermal energy, given by Eq. 5^38^.

5 $$\beta =\frac{-{B}^{2}V\left({\chi }_{r}+{\Delta }_{\chi }{\cos }^{2}\varphi \right)}{2{k}_{B}T}$$

Here, the diamagnetic anisotropy ${\Delta }_{\chi }$ is quantified as the difference along the axial and radial magnetic susceptibilities (${\chi }_{a}$ and ${\chi }_{r}$, respectively) of the species, ${\Delta }_{\chi }={\chi }_{a}-\,{\chi }_{r}$, *φ* is the angle between the symmetry axis and the external magnetic field direction, ${k}_{B}$ is the Boltzmann constant, and *T* is the temperature. Examples of biomolecular assemblies with anisotropic magnetic susceptibilities include cell cytoskeletal polymers such as actin filaments^39^ and microtubules^40^, lipid bilayer of cell membranes^41^, and polynucleotide chains^42^.

### The radical-pair mechanism arising from an interplay between the Zeeman effect and spin-orbit coupling in a biomolecular assembly

6 $$\Delta E={g\mu }_{B}B$$

Under the influence of an external magnetic field, an electronic triplet energy level “splits” into additional levels based on the orbital angular momenta available to the electrons in the species outermost electronic energy level (the Zeeman effect). The energy gap between these newly created levels is directly proportional to the intensity of the magnetic field, given by Eq. 6.

Here, *g* is the g-factor (~2 for a free electron), ${\mu }_{B}$ is the Bohr Magneton (9.274 × 10^−24^ J T^−1^), ∆*E* is the energy gap between the newly split energy levels relative to the original (unperturbed) energy level without the SMF stimulation. The coupling between the radical pair’s spin angular momentum and orbital angular momentum (spin–orbit coupling) causes the population of the singlet and triplet states of a biochemical species to also change. In a typical biomolecular photoexcited state, the change in singlet-triplet population ratio caused by a <1000 mT magnetic field, due to spin–orbit coupling and the Zeeman effect, is negligible. However, when the electron pair is delocalized over multiple species (in a radical pair, for example), the value of spin-orbit coupling can be large enough to allow for a significant magnetic field-induced change in the singlet-triplet population ratio. This change can be harnessed to alter downstream reaction rates (for example, those that depend on the concentration of a species in its’ triplet state)^43–45^. Indeed, a radical-pair mechanism involving tryptophan and flavin species’ in retinal cryptochrome proteins is thought to enable magnetoreception in night migratory birds^46,47^. Altered singlet-triplet population ratios may also be harnessed to change rates for similar biochemical reactions relevant within the context of tumors^48,49^.

## The biochemical targets of SMFs

Both spatially homogeneous and inhomogeneous SMF stimulation regimes have been shown to target a variety of intracellular species, using all three mechanisms discussed in Section “The molecular mechanisms of SMF action”. In this section, we discuss the biochemical targets, associated mechanisms and relevant experimental evidence available in existing literature on SMFs across intensity regimes. We note that cell death signaling induced by chemotherapeutic drugs and radiation therapy is enhanced by SMF stimulation, indicating its promise in combination with conventional cancer treatment modalities.

### Realigning collagen fibrils

Collagen fibrils align perpendicular to the field direction due to their anisotropic susceptibility, which exposes them to a SMF-induced torque. They were first shown to be susceptible to magnetic fields in 1984, when Torbet and Ronzière showed that they self-assembled almost uniaxially perpendicular to the direction of applied 1900 and 5600 mT SMFs^50^. 10,000 mT SMF stimulation for seven days also caused human glioblastoma A172 cell lines embedded in densely packed (type I) collagen gels to align perpendicular to the SMF direction (Table 1)^51^. The aligned collagen binds to glioblastoma cells through integrin-extracellular matrix adhesions, a process linked to the reorganization of microtubules along the collagen fibers^51,52^. This alignment, which is associated with microtubule reorganization, does not occur in the absence of the collagen gel, indicating that the cells’ orientation is a direct result of extending their processes along the magnetically aligned collagen axis.

**Table 1:** The influence of SMF stimulation on cancerous cell function at different intensities and exposure durations.

Intensity (mT)	Duration of Stimulation (H)	Type of Magnet	Cell Type	Impact on cell function	Reference
<0.0002; GMF (control sample) =39.4 ± 3.6 μT; GMF (Geomagnetic field) (control sample including CuZn-SOD) = 29.9 ± 2.85 μT	48	A 12-layer permalloy magnetic shielding chamber (MSC) (with outer Al layer) was placed inside incubator to reduce the geomagnetic field. GMF control samples were cultured on a plastic shelf located outside the MSC.	Human neuroblastoma SH-SY5Y cell line	Increased proliferation by reducing the cellular ROS levels (H_2_O_2_); antioxidation effect.	^124^
0.0002 (Shielded GMF);(control sample) = 0.5 mT; (Normal GMF)	Observation at different periods of exposure 24, 28, and 48.	A 12-layer permalloy magnetic shielding chamber (with outer Al layer) was placed inside incubator to reduce the geomagnetic field.	Human neuroblastoma SH-SY5Y cell line	Magnetic shielding induced a time-dependent acceleration of cell proliferation; Significant enhancement in G1-phase progression and cell cycle advancement was observed within the first 0–24 h, whereas after 28 h of exposure, cell cycle progression and proliferative activity were no longer significantly different from GMF control cells and continuous 48 h exposure markedly enhances the proliferation of human neuroblastoma SH-SY5Y cells.	^197^
0.0005–0.6	96	Two sets of Helmholtz coils were made of 1.63 mm wire placed inside a water-jacketed incubator to produce homogeneous flux varied within ±5%.	Human fibrosarcoma HT-1080 cell line	Biphasic Growth Response observed: Inhibition: Growth significantly decreased at 0.0005 (shielded) and 0.6 compared to control (0.045). Acceleration: Growth significantly increased at 0.3 and 0.4 ROS Balance: H₂O₂: Non-linear response; decreased at 300 µT (matching peak growth) but increased at 600 µT (matching inhibition). NO & Superoxide: Decreased with increasing field. Oxidative Stress: generally increased with field intensity. Increased SMF also caused depolarization and pH decline (altering the electrochemical gradient). Direction Dependence: Orientation specific effects were observed in the aforementioned parameters.	^115^
20, 30	96	SMF Generator that contains 40 cm solenoid with 1800 loops of 2.5 mm copper-coated wire to generate homogeneous flux.	HeLa human cervical cancer cell line	Proliferation rate decreased by 1-fold at 20 mT; Significant decrease in viability and proliferation; reduced IC₅₀ indicating enhanced susceptibility to cell death.	^206^
35-120	168	Twelve solid magnets (5.1 × 2.5 × 0.3 cm) were used, with six magnets placed on each of two separate steel plates. The cells are positioned between the two steel plates to generate heterogeneous flux.	LIDRU-80 cell line	SMF exposure did not affect the initial attachment of LIDRU-80 melanoma cells; However, it significantly inhibited their subsequent proliferation, resulting in approximately a 20% reduction in cell number compared to controls by day.	^153^
100	0.22 (13 min)	Toroidal electromagnet (80*130*25 mm) with pole gap (25*15*25 mm), generating a homogeneous flux varied within ±5%.	Human leukemia HL-60 cell line	No change in [Ca^2+^]; Altered ATP-induced intracellular Ca²⁺ responses, particularly following GSH (glutathione) depletion.	^121^
150; GMF control samples were 65:08 ± 7:18 μT	48–72	Rectangular NdFeB magnet	Mouse breast cancer 4T1 cell line	Increased proliferation; Accelerated cell proliferation while significantly inhibiting cell migration. Shortened telomere length, reduced telomerase activity and TERT expression, likely via upregulation of E2F1-indicating suppression of telomerase function.	^136^
260–330 [Gradient- 2090 mT/m]	72	Cells were placed outside the cylindrical superconducting magnet where flux density ranged 200–400 mT with radial gradient of 2090 mT/m inside a copper incubator maintained at 37 *°*C.	Human cervical cancer HeLa cell line and MCF-7 cell line	No effect on proliferation; Modulated biomechanical properties of MCF-7 and HeLa cells, likely through cytoskeletal reorganization, and reduced cell adhesion, associated with altered membrane ultrastructure.	^233^
500	72	Cylindrical NdFeB permanent magnets.	Human liver cancer Hep G2 cell line	No change in [Ca^2+^]; Synergistically enhanced capsaicin’s anticancer effect on HepG2 cells by increasing TRPV1-mediated calcium influx, promoting mitochondria-dependent apoptosis through elevated Bax/Bcl-2 ratio and caspase-3 activation.	^79^
500	504–672	A C-shaped 3.75 cm^2^ soft iron bar with 2 neodymium-iron-boron (NdFeB) magnets at the pole pieces, forming a 4.1 cm air gap between the two ends.	GH3 rat pituitary gland cell line	Proliferation decreased by 51% with an increase in cell diameter; One-week SMF exposure caused a non-significant 22% reduction in cell growth, which recovered within a week, whereas 4-week exposure led to a significant 51% growth inhibition. Prolonged exposure to a 0.5 T static magnetic field decreases GH3 cell proliferation and increases cell size in a time-dependent and reversible manner, likely through altered intracellular Ca²⁺ levels that modulate actin cytoskeleton reorganization.	^139^
1000	72–120	Permanent magnet: The maximum magnetic field intensity was at the top center of its surface, where the culture plate was placed. (homogeneous flux)	CNE-2Z human nasopharyngeal carcinoma cell line	Inhibited cell proliferation; Inhibited the Akt/mTOR pathway in CNE-2Z cells; Enhanced the effects of mTOR inhibitors on pS6K and p4EBP1 and prevents feedback EGFR reactivation.	^142^
1000	48	Permanent magnet (5 × 5 × 5 cm); the petri dish was placed directly on the top center of the magnet. (homogeneous flux)	CNE-2Z human nasopharyngeal, HCT116 human colon, A431 human skin, A549 human lung, MCF-7 human breast, PC3 human prostate, and EJ1 human bladder cancer cell lines	Inhibited cell proliferation; Enhanced the anticancer efficacy of Akt inhibitors (BEZ-235 and MK2206) in CNE-2Z cancer cells. At high cell density, SMF reduced cell numbers in 6 out of 7 human solid cancer cell lines, whereas no growth inhibition was observed at low density. The growth inhibition can be explained through inhibition of the phosphorylation/activation of EGFR and Akt in high-density cancer cells.	^60^
1000	12	Permanent magnet	HeLa human cervical, MCF-7 human breast and HCT116 human colorectal carcinoma cell lines	Decreased cell growth; Disruption of mitotic spindle organization, mitotic arrest, and enhanced sensitivity to chemotherapeutic agents (5-FU and Taxol), leading to reduced proliferation.	^88^
1000	48	Permanent magnets	Human glioblastoma U251, Human colon epithelial carcinoma HCT116, Human hepatocellular carcinoma HepG2, Human gastrointestinal stromal GIST-T1, Human nasopharyngeal CNE-2Z, and Human bladder EJ1	Decreased intracellular [ROS] by 10–20% which authors explain through the phenomenon of SMF acting as a “physical antioxidant” likely by interacting with the electron transport chain in mitochondria to reduce electron leakage and superoxide formation.	^203^
1500 (with gradients up to 200 T^2^/m at 0.1 mm)	96	Sm-Co permanent magnet with Au-coated iron needle, producing non-homogeneous SMF	Human cervical cancer HeLa cell line	No change in morphology or colony formation; Altered cell growth rate with inhibitory effects depending on field gradient and exposure time.	^238^
4700	12, 24, 48 and 72	A superconducting MRI magnet with the gradient coils removed, housed within a copper-shielded enclosure.	Human malignant melanoma PS1273 cell line	No effect on cell number or its viability; After 48 h of exposure to a 4700 mT SMF, only up to 3.5% of melanoma cells remained attached compared to untreated controls, indicating a strong inhibitory effect on cell adhesion. significant reduction in tumor cell adhesion to the substrate by disrupting cytoskeletal organization and focal adhesion integrity, leading to cell rounding and decreased attachment strength without inducing direct cytotoxicity or loss of cell viability.	^146^
12,000	48	Non-refrigerant superconducting magnet housed in an incubation chamber.	MNNG/HOS, U-2 OS, and MG63 human osteosarcoma cell lines	Inhibited cell proliferation, Intracellular ROS levels were reduced, iron metabolism was altered, and cell cycle progression was suppressed accompanied by downregulation of CDK1 and cyclin B1 and alterations in G1/S regulatory proteins (CDK2, CDK4, cyclins D1 and E1), contributing to antiproliferative effects.	^127^
27,000	4 (acute exposure during mitosis)	Water-cooled 27,000 mT magnet housed in coaxial non-magnetic stainless-steel tubes.	CNE-2Z human nasopharyngeal cancer cell line	Cell number decreased by 40%; Altered mitotic spindle orientation and morphology; disrupts spindle alignment during cell division, potentially impairing accurate chromosome segregation and affecting proliferation.	^40^
10,000	1	Magnet with integrated CO_2_ incubator	Human glioblastoma A172 cell line (embedded in type I collagen)	Oriented perpendicular to SMF when embedded in collagen, No change in orientation without collagen. Induced directional orientation and alignment of cells and cytoskeletal structures without affecting cell viability or proliferation. In A172 cells embedded in collagen gel, exposure to a 10,000 mT SMF aligned β-tubulin–labeled microtubules parallel to the magnetically oriented collagen fibers.	^51^

### Disrupting the cell membrane

Within the cell membrane, the anisotropic magnetic susceptibility of constituent membrane proteins and the phospholipid bilayer causes the emergence of an SMF-induced torque in both of their structures. In rodent pituitary cancer GH3 cell lines, 120 mT SMF stimulation increased the time taken for voltage-activated Ca^2+^ channels to open when subjected to a range of bias voltages^53^. Another set of experiments on the same cell lines showed that 125 mT SMF stimulation for 150 s decreased peak Na^+^ current through voltage-gated Na^+^ channels by 5%^54^. Such changes in the current–voltage relationships of Ca^2+^ and Na^+^ ion channels were proposed to arise from SMF-induced disruption in the phospholipid bilayer. Moreover, SMF-induced disruption in the structure and function of the cell membrane can also potentially alter the membrane potential. For instance, 0.6 mT SMF stimulation for 5 min increased membrane potential in human leukemia U937 cell lines, but had the opposite effect in Jurkat cell lines, respectively^55^. The implications of this altered membrane potential are further discussed in Section “Increasing intracellular Ca^2+^ flux" but the underlying reasons behind such a difference in response to SMF stimulation are unclear (Section “The physiological parameters that govern tumor response to SMF stimulation").

In addition to the phospholipid bilayer, SMFs can also cause structural changes in membrane-embedded receptor proteins. 700–1000 mT SMF stimulation for a few minutes to 1 h inhibited the activity of purified Epidermal Growth Factor Receptor (EGFR) kinase domains in solution^56^. EGFR is frequently overexpressed in a variety of tumors, and its activation is associated with an increase in cancer cell proliferation, invasion, metastasis, and angiogenesis^57,58^. EGFR kinase domains reoriented to the magnetic field direction, preventing EGFR dimerization (crucial for trans-autophosphorylation and subsequent downstream signaling). 500–1000 mT SMF stimulation for 3 days inhibited proliferation in EGFR-expressing cell lines (including human colon cancer HCT116, nasopharyngeal carcinoma CNE-2Z, and cervical cancer HeLa), but not in the Chinese Hamster Ovary (CHO) cell lines, which lack endogenous EGFR expression. Previous work on CHO cells using higher intensity SMF stimulation (1000–10,000 mT for 3 days) also showed proliferation inhibition only when transfected with EGFR^55,59^. This modality of targeting EGFR signaling by SMF stimulation has been previously proposed to be density-dependent in CHO cells^60^. Moreover, an investigation demonstrated the disruption of the EGFR pathway In Vivo, shedding light on the antagonistic effect SMF stimulation may play in EGFR-expressing carcinomas in tandem with an anti-EGFR antibody like cetuximab^61^.

In addition to the cell membrane, the mitochondrial membrane is susceptible to SMF-induced disruption. For example, in several rat and human cell lines, 1000 and 1130 mT SMF stimulation for 6 h increased mitochondrial membrane potential^62^. Because the mitochondrial membrane potential drives the activity of the ATPase proton pump, SMF stimulation resulted in increased intracellular ATP concentration. This idea of a surge in ATP concentration has also been displayed for immune cells (Table 3)^63^. Moreover, in human neuroblastoma SH-SY5Y cell lines, 2.2 mT SMF stimulation for 24 h decreased the mitochondrial membrane potential by 30%^64^. The increase in proportion of β-sheet content relative to α-helix content in F_0_F_1_ ATPase possibly caused this decrease in mitochondrial transmembrane potential^64^. Collectively, these findings show that SMF stimulation can influence cellular bioenergetics through altered intracellular ATP concentrations. Intracellular ATP concentrations dictate overall cellular metabolic activity, and may partly explain SMF-induced alterations in cellular metabolic activity^65^.

**Fig. 3:**
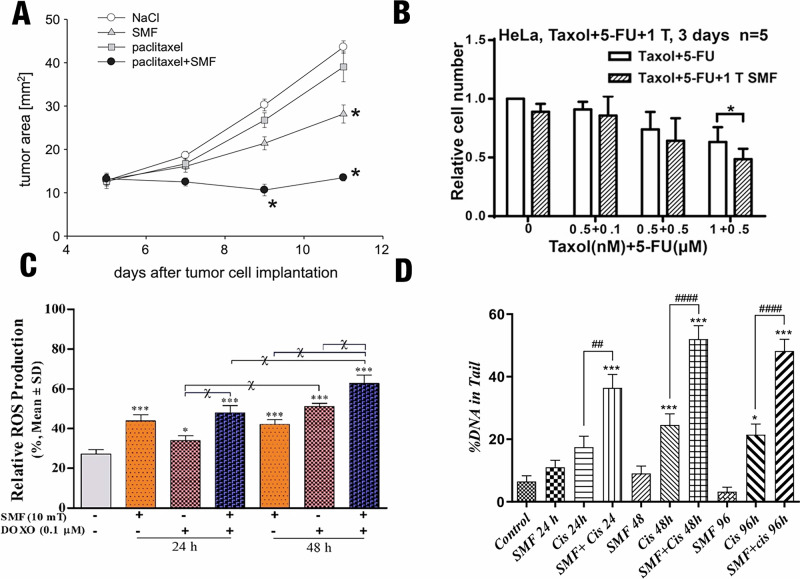
Synergistic effects of SMFs with chemotherapeutic drugs In Vitro synergy. **A** In A-Mel-3-tumor-bearing hamsters, tumor area was lowest in the group treated with paclitaxel (Taxol; 5 mg/kg body weight) + 587 mT SMF compared with NaCl, SMF alone, or paclitaxel alone. Significant tumor growth delay was evident after the second treatment and persisted until day 11. The stars represent significant differences (*p* < 0.05 vs all other groups, Kruskal–Wallis rank sum test) between tumor area at measured points and all other groups. Figure adapted from ref. ^69^ Copyright Elsevier, 2014. **B** In human cervical cancer HeLa cells, 1000 mT SMF stimulation increased the cytotoxicity of 5-Fluorouracil (FU) and Taxol, reducing viable cell numbers by ~10–20% compared with chemotherapy alone (*p* < 0.05, Student’s *t* test, *n* = 5). Bars represent mean ± SD. Figure adapted with permission from ref. ^88^ Copyright Elsevier, 2016. **C** In human breast cancer MCF-7 cells, 10 mT SMF stimulation increased doxorubicin-induced intracellular [ROS] measured by DCFDA flow cytometry. Measured [ROS] rose from 27.2% in sham to 48.0% (24 h) and 62.8% (48 h) in SMF + doxorubicin, significantly higher than either monotherapy (*p < 0.01 and **p < 0.001, one-way ANOVA with post-hoc test, *n* = 3). Figure reproduced with permission from ref. ^114^ Copyright Nature Publishing Group, 2018. In Vivo *synergy:* **D** In A2780 cells, 15 mT SMF increased cisplatin-induced DNA strand breaks, as shown by a higher percentage of DNA in comet tails (**p < 0.001 vs control; ^##^*p* < 0.01 and ^###^*p* < 0.001 vs cisplatin, *n* = 3). Figure reproduced with permission from ref. ^138^ Copyright Nature Publishing Group, 2024.

In many cases, SMF-induced cell membrane disruption appears to enhance the effectiveness of chemotherapeutic drugs^66–68^. In human leukemia-derived K562 cell lines, 9 mT SMF stimulation for 12–24 h increased membrane permeability and improved the uptake of chemotherapeutic drugs such as taxol, doxorubicin, cisplatin and cyclophosphamide (Table 2)^68^. In A-Mel-3-tumor-bearing hamsters In Vivo, 587 mT SMF stimulation for two h on days 5, 7, and 9 after tumor implantation increased the permeability of tumor microvessels, which are responsible for supplying nutrients and oxygen to tumor masses^69^. The use of this SMF stimulation and paclitaxel (5 mg/kg body weight) separately decreased tumor growth, but when used in combination, their application stopped tumor growth altogether (as measured by tumor area; Fig. 3A). However, the synergistic contribution of SMF stimulation may only be limited to compromising the plasma membrane, without influencing overall cell viability^70^. Collectively, these results show that SMFs may work effectively in tandem with chemotherapeutic drugs to enhance their efficacy for tumor treatment in human subjects.

### Increasing intracellular Ca^2+^ flux

Ca^2+^ is a crucial secondary messenger that controls cell survival, growth, and apoptosis^71,72^. A torque on the phospholipid bilayer of the cell membrane, arising from its anisotropic magnetic susceptibility (Section “The molecular mechanisms of SMF action"), also causes Ca^2+^ ion channels to be structurally disrupted as they rotate in response to a magnetic field, causing a major influx of Ca^2+^ into the cell^53,73^. The Ca^2+^ influx may also be explained through coincident alteration of membrane polarization, although a definitive causation has not yet been established^55^. However, L-type Ca^2+^channels are sensitive to membrane polarization, allowing them to possibly act as links between SMF-induced changes in membrane polarization and Ca^2+^ influx.

In human histiocytic lymphoma U937 cell lines, 6 mT SMF stimulation for 1–4 h enhanced Ca^2+^ influx and decreased chemical (etoposide)-induced apoptosis^74^. Increasing SMF intensity increased the percentage of apoptotic cells. Additionally, in human primary glioblastoma cells, 6 mT SMF stimulation for 20 min increased the intracellular [Ca^2+^] from 124 ± 4 nM to 233 ± 43 nM, decreasing heat shock and chemical (etoposide)-induced apoptosis^75^. Moreover, in human histiocytic lymphoma U937 cell lines, 6 mT SMF stimulation for 4 h increased intracellular [Ca^2+^] and decreased apoptosis.

Conversely, SMF stimulation in the 1–10 mT range can also induce apoptosis over longer durations. For example, in T-lymphoblastoid Jurkat E6.1 cell lines, 6 mT SMF stimulation for >36 h increased the Ca^2+^ influx, caused cell cycle arrest and induced apoptosis via a *p*53-independent pathway^76^. Similarly, in human liver cancer HepG2 cell lines, 6 mT SMF stimulation for 4–24 h increased the intracellular [Ca^2+^], increasing apoptosis with stimulation time^77^. Increased intracellular [Ca^2+^] also led to actin filament reorganization, which ultimately changed cell shape and cell surface morphology.

In human liver cancer HepG2 cell lines, 500 mT SMF stimulation for 72 h in tandem with 25 µM and 50 µM capsaicin (which has anti-cancer activity^78^, and is well known to bind and activate the opening of TRPV1 Ca^2+^ ion channels) caused a surge in intracellular [Ca^2+^], which resulted in a dramatic increase in Bax/Bcl-2 ratio, which led to enhanced apoptosis through mitochondrial permeabilization (Tables 1 and 2)^79^. Although the aforementioned studies consistently show that SMF stimulation causes an increase in intracellular [Ca^2+^], the question of how it influences the spatial distribution and influx of other ions, and the activity of ion channels, is not well answered and should be the focus of future research (Section “Future outlook and summary”). Collectively, these studies indicate that SMF-induced increase in [Ca^2+^] can profoundly influence signaling, eventually altering cell viability.

### Reorganizing the cytoskeleton

The cytoskeleton is a network of protein polymers that collectively perform structural roles in the cell. The three main polymers of the cytoskeleton are (a) microtubules, which are hollow cylindrical tubes of the globular protein tubulin; (b) actin filaments, which are double helical polymers of protein actin; and (c) intermediate filaments, which are a group of protein polymers that coordinate the mechanical forces in both the nucleus and the cytosol^80^. Cytoskeletal polymers work together to orchestrate cell division^81,82^, generate and coordinate intracellular forces required for cell movement^81,83^, maintain cell shape^84^ and form a closely interacting meshwork for intracellular macromolecular transport^85^.

SMF stimulation reorients microtubules such that they align parallel to the SMF direction, because the lattice-like head-to-tail arrangement of tubulin dimers results in a highly anisotropic susceptibility. Early work indicated that microtubules self-assembled from tubulin in buffer solution reoriented along the magnetic field direction, upon relatively weak intensity (20 mT) SMF stimulation^86^. It was revealed later that, upon high intensity (11,000 mT) SMF stimulation too, microtubules self-assembled in buffer solution along the magnetic field direction^87^. For instance, in human nasopharyngeal cancer CNE-2Z cell lines, 27,000 mT SMF stimulation for 4 h altered the morphology and orientation of spindle microtubules, such that they reoriented closer to the magnetic field direction (Table 1)^40^. However, the change in orientation and morphology of the mitotic spindle was influenced more by the anisotropic susceptibility of the chromosomes than that of the microtubules. In human cervical cancer HeLa cell lines, 1000 mT SMF stimulation for 7 days led to a 2.5-fold increase in multipolar abnormal mitotic spindles, possibly through reorientation during mitosis^88^. Due to the critical role played by microtubules in cell proliferation, they are well-recognized as targets of several chemotherapeutic drugs, including taxanes and *Vinca* alkaloids^88^ In human colon cancer HCT116, nasopharyngeal carcinoma CNE-2Z, cervical cancer HeLa, and breast cancer MCF7 cell lines, 1000 mT SMF stimulation for 3 days enhanced the antitumor efficacy of 0.5 µM 5-Fluorouracil and 0.5 µM 5-Fluorouracil +1 nM Taxol evinced by decreasing the cell number (Fig. 3B) (Table 2)^88^. Thus, it is likely that additional reorganization of the microtubule cytoskeleton by SMF stimulation will improve anticancer drug activity In Vivo.

Actin filament networks also undergo reorganization upon low-intensity SMF stimulation. In human liver cancer Hep G2 cell lines, 6 mT SMF stimulation for 24 h led to the formation of lamellar-shaped microvilli on the cell surface, where bundles of actin filaments were observed^77^. In human leukemia U937 and cervical cancer HeLa cell lines, 6 mT SMF stimulation for 24 h also resulted in dramatic morphological changes, which were explained partly by actin filament reorganization^89,90^ In addition to their reorganization, the density of actin filaments is also influenced by low-intensity magnetic fields: In human neuroblastoma SH-SY5Y cell lines, hypomagnetic <0.0002 mT SMF stimulation for 48 h decreased actin filament density, leading to impaired cell migration, cell adhesion, and motility^27^. The response of actin filaments to SMF stimulation can have multiple explanations: First, increased ROS accumulation (which is also a consequence of low-intensity SMF stimulation; Section “Accumulating ROS") may oxidize surface-exposed redox-active residues in actin, causing structural changes in the polymer, which ultimately lead to depolymerization^91–93^. Second, an increase in intracellular [Ca²⁺] (also a consequence of SMF stimulation, Section “Increasing intracellular Ca^2+^ flux") can activate regulatory proteins such as the gelsolin family, which, upon Ca²⁺ binding, recruit, sever, and cap actin filaments, preventing further polymerization^94,95^. Third, the intrinsic anisotropic susceptibility of actin filaments can cause them to realign upon SMF stimulation, causing reorganization in the cytoskeleton. Detailed modeling studies of the influence of SMF stimulation on the actin cytoskeleton are yet to be performed. Notably, the influence of SMF stimulation on intermediate filaments has not yet been investigated (Section “Future outlook and summary”).

### Accumulating ROS

ROS can be produced in the mitochondria as a byproduct of the electron transport chain (ETC), when oxygen molecules undergo reduction to form ROS such as hydrogen peroxide (H_2_O_2_), hydroxyl (${\left[\mathrm{OH}\right]}^{\cdot }$), and superoxide (${\left[{{\rm{O}}}_{2}\right]}^{\cdot -}$)^96,97^. ROS can also be produced in the endoplasmic reticulum as a consequence of oxidative folding^98,99^. Exposure to electromagnetic radiation with wavelengths that excite ground-state molecular oxygen (which is in the triplet manifold) into its highly reactive and long-lived singlet state (^1^O_2_) can also produce ROS in the cytosol^100–103^. Singlet oxygen then reacts with biomolecules to produce other ROS, ultimately causing redox stress to the cell^48,104,105^. This mechanism is harnessed by photodynamic therapy to ablate tumor tissue, which is crucial for skin cancer treatment. Although ROS were previously considered to harm cell health, evidence has emerged showing that ROS can also influence the cell positively. For example, during the “respiratory burst”, NADPH oxidases in the cell membrane of phagocytic cells inject ROS into the extracellular matrix, damaging pathogenic stimuli through redox stress as part of immune defense^106^. ROS can also play crucial signaling roles for the benefit of the host cell, such as activating immune and inflammatory responses through the transcription factor NF-κB^107^, initiating cell motility by activating the protein SSH-1L^108^ and regulating growth signaling through the kinase hub Akt^109–111^.

### The models of ROS generation by SMF stimulation

SMF-induced ROS accumulation has been reported across several cancer cell lines at different SMF intensities and exposure durations, including human leukemia U937^112^, THP-1^113^, human breast cancer MCF-7^114^, HT-1080 human fibrosarcoma cell line^115^, cervical cancer HeLa^116^, ovarian cancer HO8910 and SKOV3^117^, lung adenocarcinoma A549^37^, double-strand break repair-deficient XRS-5 human–hamster hybrid (A^L^) and mitochondria-deficient (*ρ*^0^A^L^) cell lines^118^. Beyond in vitro cell lines, there have been reports indicating SMF-induced ROS accumulation within In Vivo models (Table 3)^37,117^. Reported 2.2 mT SMF stimulation for 24 h led to an increase in ROS concentration in human neuroblastoma SH-SYSY cell lines, also causing a decrease in mitochondrial membrane potential, suggesting that impaired electron flow in the mitochondrial membrane was responsible for intracellular ROS accumulation^64^. Another study reported coincident changes in ATP biosynthesis and ROS accumulation as a result of SMF stimulation (8500 mT for 3 h) in human hamster hybrid (A^L^) cells, mitochondria-deficient (p^0^A^L^) cells, and double-strand break repair-deficient (XRS-5) cells^118^, strengthening the premise of SMF-induced ROS accumulation via mitochondrial membrane disruption.

#### Decrease in antioxidant concentration

Biomolecular antioxidants react with ROS to produce stable products, mediating the delicate balance between cell signaling for maintaining health and that which initiates cell death^119,120^. Thus, SMF-induced increase in ROS concentrations leads to a concomitant decrease in the concentration of antioxidants. SMF-induced changes in ROS concentrations, achieved by shifting radical pair recombination yields through the radical-pair mechanism, can impact oxidative stress and signaling pathways. For example, in human leukemia U937 cell lines, 6 mT SMF stimulation for 2 h increased ROS concentration, as expected from the radical-pair mechanism. Additionally, a concomitant decrease in concentration of the antioxidant glutathione (GSH), which gets consumed while reacting with ROS, was observed^112^. The premise of GSH countering SMF-induced effects is strengthened by the observation that in HL-60 cells, SMF stimulation (100 mT for 13 min) led to altered ATP-induced intracellular Ca²⁺ responses, particularly following GSH depletion (Table 1)^121^. Similarly, in cancerous gastric tissue extracted from human subjects, 100 mT SMF stimulation for 1 h decreased concentrations of the antioxidants such as superoxide dismutase and glutathione peroxidase^122^. Consistent with SMF-induced ROS accumulation^123^, the study also reported increased concentration of the lipid peroxidation marker malondialdehyde. However, investigations have also revealed a decline in the concentrations of antioxidants like superoxide dismutase^122,124^ and glutathione peroxidase^122^ upon SMF stimulation. The diverse responses of different antioxidants to the same SMF treatment indicate that there may be specific ROS species that are selectively targeted by SMF stimulation. Additionally, alongside antioxidant and anti-cancer drugs like flavonoid compounds, SMF stimulation has demonstrated a variety of antagonistic effects, again emphasizing that more work is required to precisely understand the interface of the SMF stimulation, antioxidant-ROS activity, and the radical-pair mechanism mechanistically relying on ROS increment for targeting tumor cells^125^.

#### The iron equilibrium

SMF stimulation has been shown to modulate the Fe²⁺/Fe³⁺ redox equilibrium by facilitating [Fe^2+^] membrane transport. SMF-induced increase in intracellular [Fe^2+^] can be achieved through (A) the upregulation of divalent metal transporter 1 (DMT1), responsible for maintaining iron homeostasis by mediating transmembrane uptake of Fe^2+^, and (B) downregulation of ferritin heavy chain 1 (FTH1), which oxidizes Fe^2+^ into Fe^3+^, sequestering excess complexes to prevent iron-induced toxicity^126^. Increased intracellular [Fe^2+^] in turn increases local ROS concentrations via the Fenton reaction $({{\rm{Fe}}}^{2+}+\,{{\rm{H}}}_{2}{{\rm{O}}}_{2}\to {{\rm{Fe}}}^{3+}+{\rm{OH}}+{{\rm{OH}}}^{-})$, which ultimately leads to proliferation inhibition and triggers G2/M cell cycle arrest. Thus, cell fitness, survival, and death are subject to its ROS concentration, which can be mediated by the iron equilibrium inside the cell. The experimental data presented in this context obscure a clear understanding of how SMF influences iron equilibrium in cancer cell lines. For instance, 12,000 mT SMF stimulation for 24–48 h inhibits the proliferation of human osteosarcoma MNNG/HOS, U2OS, and MG63 cell lines via intracellular iron accumulation, leading to ROS-associated cell cycle arrest (Table 1)^127^.

On the other hand, another report mentions that MCF-7 cells exhibit a significant decrease in intracellular concentration of iron (Fe (II) and Fe (III)) in the presence of 10 mT SMF (for 24 and 48 h)^114^. A steep increase in the ROS concentration was also observed, alongside a decline in intracellular iron content. Another interesting investigation reveals an uptick in intracellular Fe^2+^ concentration in K7M2 and MG63 osteosarcoma stem cells (OSCs) upon prolonged exposure (7 days) to 200–400 mT. This was concurrent with a decrease in FTH1 expression^128^. Notably, there was a temporary increase in intracellular ROS concentration, but the cells adapted over time, resulting in no significant change in ROS levels in the long term (7 days). On balance, the exposure promoted the self-renewal capacity of the OSCs and thus aided cancer proliferation. Consequently, we believe that the interface of SMFs, iron homeostasis and ROS accumulation in cancer cells warrants more thorough investigation^114^.

#### The consequences of SMF-induced ROS increase

SMF-induced ROS concentration increase can lead to a variety of downstream consequences. For example, in human ovarian cancer HO8910 and SKOV3 cell lines, 100–500 mT SMF stimulation for 24 h caused an increase in ROS concentrations, resulting in decreased cell invasion and migration (measured by wound healing)^117^. Increased ROS also downregulated stemness-related genes, including *Sox2*, *Nanog*, *C-myc, CD44*, and *CD133*. The decrease in stemness lowered the ability of cancer cells to self-renew, lowering metastasis as well as tumor recurrence^117^. In lung cancer cell-bearing male BALB/c mice, 9400 mT SMF stimulation for 88 h increased ROS concentration, which in turn inhibited DNA synthesis and increased the concentration of the protein p53, which is strongly associated with tumor growth inhibition^37,129^. In human leukemia THP-1 cells, 1.2–2.4 × 10^5^ mT/cm SMF stimulation for 24 h resulted in triggered ROS accumulation, which increased cell swelling and triggered apoptosis^113^. Lower-intensity (1200 mT/cm) SMF stimulation did not induce sufficient stress to drive cells towards apoptosis. In human lung adenocarcinoma A549 cell lines, 9400 mT stimulation for 24 h nearly doubled ROS concentration, notably increasing the concentration of the protein p53 without triggering apoptosis^37^. Collectively, these findings indicate that SMF stimulation likely increases ROS accumulation, impairing cell functioning in a variety of ways. An excellent review on the influence of SMF stimulation on tumors can be found in ref. ^130^.

In addition, SMF-induced tissue damage by ROS has been shown to enhance the efficacy of chemotherapy in different cell lines. For instance, in human glioblastoma A172 cell lines, SMF stimulation of 5 mT for 96 h in combination with 200 μM temozolomide significantly increased ROS production and upregulated p53 expression, leading to enhanced cytotoxicity (Table 2)^131^. Similarly, 10 mT SMF stimulation for 48 h in combination with 0.1 μM doxorubicin doubled ROS production *via* the Fenton reaction in human breast cancer MCF-7 cell lines (Fig. 3C) (Table 2)^114^. Cancer treatment modalities such as photodynamic therapy and radiation therapy induce ROS accumulation in tumor tissue, causing redox stress, which ultimately leads to cell death^132–135^. Looking forward, these findings suggest that SMFs can synergize with chemotherapeutic drugs to amplify such ROS-mediated cancer cell death, demonstrating their potential in combination therapy (Section "Future outlook and summary”)^114,131^.

### Damaging DNA integrity

SMF stimulation influences the stability of DNA polymers by causing structural damage through (A) ROS accumulation, which causes DNA nucleobases to undergo redox reactions, (B) by causing rotational stress on DNA due to the Lorentz force, and (C) by casing rotation of DNA polymers arising from their anisotropic magnetic susceptibility.

At lower intensities (<1000 mT), SMF stimulation induces ROS accumulation, causing DNA nucleobases to undergo redox reactions. In human T-lymphoblastoid Jurkat E6.1 cells, 6 mT SMF stimulation for 24 h caused ATM and E2F1 phosphorylation, triggering DNA repair and activating apoptotic signaling pathways^76^. In mouse breast cancer 4T1 cell lines, 150 mT SMF stimulation for 72 h decreased both telomere length and telomerase activity by influencing the expression of telomerase reverse transcriptase (TERT), which is responsible for maintaining telomere length (Table 1)^136^. SMF stimulation also caused an elevation in the levels of the transcription repressor E2F1, which inhibits TERT and thus regulates the cell cycle. It was also observed that these SMFs caused the migratory efficiency of these cells to decrease by 71.6%. The exact mechanism by which DNA and DNA expression are altered by these SMFs is still unclear but likely involves ROS accumulation and associated redox reactions with DNA nucleobases.

High intensity (>1000 mT) stimulation results in an SMF-induced torsion on DNA polymers. Torsion arises from the angular component of the Lorentz force^137^, which itself emerges from their mechanical rotation during replication and transcription, and their negative surface charge at physiological pH. In human lung adenocarcinoma A459 cell lines, higher intensity (9400 mT) SMF stimulation for 24 h decreased DNA synthesis by 10–20%^37^. SMF-induced torsion from the Lorentz force was thought to be responsible for decreased DNA synthesis on SMF stimulation in a manner depending on the direction of stimulation. The SMF-induced torsion contributed together with ROS accumulation to cause this effect, also leading to enhanced p53 expression and elevated the levels of pCDK1(Y15) and cyclin B to cause G2 cell cycle arrest. Arrested DNA synthesis by SMF stimulation has also been reported for other cell lines^37^. For example, in human colorectal carcinoma HCT116 and LoVo and lung carcinoma A549 and PC9 cell lines, 1000 mT SMF stimulation for 8 h resulted in a decrease in DNA synthesis by 5–15% when the magnetic field direction was directed upwards of the cell culture plane (no change in DNA synthesis was observed for magnetic fields directed in the downward direction; Section 5 “Unraveling the discrepancies in SMF-induced tumor reponse”)^137^. A possible explanation was thought to be given by the angular velocity of negatively charged DNA polymers, as they rotate during translation and replication, exposing them to an SMF-induced Lorentz force.

Higher intensity SMF stimulation can also reorient DNA polymers perpendicular to the magnetic field direction because of the polymer’s anisotropic magnetic susceptibility^42^. In human nasopharyngeal carcinoma CNE-2Z cell lines, 27,000 mT SMF exposure for 4 h resulted in a significant rise in the fraction of spindles with chromosomes oriented parallel to the magnetic field direction in metaphase, but aligned perpendicular to the magnetic field direction in prometaphase (Table 1)^40^. However, lower SMF intensities (such as 9000 mT) required 3 days to cause similar reorientation.

As with the other biochemical targets of SMF stimulation, DNA damage is also enhanced when used in combination with chemotherapeutic drugs (Table 2). For example, in human ovarian cancer A2780 cell lines, 15 mT SMF stimulation for 96 h in tandem with cisplatin (which induces toxic DNA lesions through inter-strand and intra-strand crosslinking) at its IC_50_ concentration enhances DNA damage as compared to unaccompanied cisplatin administration (Fig. 3D). In the same cell lines and SMF spatiotemporal profile, standalone SMF stimulation led to negligible DNA damage^138^. SMF stimulation also upregulated expression of apoptotic genes and elevated expression of CTR1, which mediates cisplatin entry into the cell. In human myelogenous leukemia K562 cell lines, when 8 mT SMF stimulation for 12 h was coupled with 25 μM adriamycin (doxorubicin), increased DNA lesions due to ROS accumulation were observed. Notably, no significant therapeutic effects were obtained through standalone treatment of either adriamycin or SMF stimulation alone^67^. Moreover, SMF stimulation can also act synergistically with DNA-damaging agents like camptothecin (CPT) to accelerate the kinetics of cell death (triggered by CPT), without influencing cell viability^70^. Thus, the amount of DNA damage done to a cell, cell viability, and the kinetics of the death are all metrics that need to be studied in reference to future work.

**Table 2:** The influence of SMF stimulation when used in combination with chemotherapy and X-ray-induced radiation therapy.

Intensity (mT)	Duration of stimulation (h)	Type of magnet	Combination therapy	Cell Type/Animal bearing tumor cell	Mechanism involved	Significant Outcome	Reference
**Chemotherapeutic drug**							
0.1	3	Merritt-like coil system consisting of three coils spaced 13 cm apart, made of coated copper wires, with 12 turns on the outer coils and 5 turns on the inner coil (homogeneous flux).	Doxorubicin (5.0 μM)	MCF-7 human Breast cancer cell line	SMF decreased DNA damage caused by doxorubicin, but no effect was observed in cytosolic or mitochondrial superoxide concentrations; No detectable influence of SMF on DNA damage response or clonogenic potential.	No impact on the efficacy of doxorubicin; Did not alter clonogenic survival of doxorubicin-treated cells, indicating no synergistic or antagonistic interaction with chemotherapy.	^207^
3	24	3 Helmholtz coils of the same radius connected in series (homogeneous flux).	Tumor necrosis factor-related apoptosis-inducing ligand (TRAIL)—a cytokine has antitumor activity (100 ng/mL)	MDA-MB-468 and T47D human breast carcinoma cell lines (TRAIL resistant cell lines)	SMF inhibited Cyclin B/Cdc2 and Cyclin A/Cdk2 complex activity, which was linked to the transcriptional repression of cyclin A and cyclin B. This decreased Cdc2 activity led to the degradation and downregulation of survivin protein levels, causing G2/M arrest and sensitizing the TRAIL-resistant breast cancer cells to apoptosis.	Synergistic effect- Significantly enhanced TRAIL-induced apoptosis, increasing cancer cell sensitivity to TRAIL without inducing apoptosis alone.	^140^
5	96	Three NdFeB magnetic parallelepipeds, coated with nickel, grade N40, Br: 1260–1290 mT, magnetized through the thickness and contained in six shelves of plexiglass structure.	Temozolomide (200 μM)	A172 human glioblastoma cell line	Decreased cell viability by 60% and increased ROS production by 151%; The MTT assay showed a modest reduction in cell viability with SMF alone (up to 8%), while temozolomide caused a pronounced decrease (up to 57%), which was further enhanced (up to 60%) when combined with SMF. Consistently, NBT analysis revealed a slight increase in free radical production with SMF (up to 8%), a substantial rise with temozolomide (up to 136%), and the highest increase (up to 151%) under combined TMZ + SMF treatment, indicating a synergistic cytotoxic and pro-oxidative effect. SMF modulates intracellular free radical (ROS) levels and alters p53 gene expression, enhancing TMZ-induced cytotoxic signaling.	Synergistic effect-Combined SMF + TMZ treatment significantly reduced cell viability, increased oxidative stress, and upregulated p53 expression compared to TMZ alone.	^131^
6	5	Magnetic disk of known intensity.	Camptothecin(0.15 μM)	HL-60 human promyelocytic leukemia cell line	Accelerated the transition from early apoptosis to late apoptosis/secondary necrosis, evidenced by a decrease in early apoptotic cells and a dramatic increase in late apoptotic/necrotic cells. The overall proportion of cells undergoing apoptosis remained unchanged.	Synergistic effect-Enhanced execution phase of apoptosis, leading to faster membrane breakdown.	^70^
8.8	8	A solenoid consisting of copper wire wound around a ring 54.7 cm long with a radius of 4 cm (homogeneous flux).	Cisplatin (10 µg/mL)	K562 human chronic myelogenous leukemia line	Decreased P-glycoprotein expression resulted in increased intracellular cisplatin concentration, which in turn resulted in decreased metabolic activity of cells. Downregulation of P-glycoprotein (MDR1), altered membrane permeability, reduced drug efflux.	Synergistic effect-Enhanced cisplatin cytotoxicity, reduced multidrug resistance, increased intracellular drug accumulation.	^66^
8.8	12	A solenoid consisting of copper wire wound around a ring 54.7 cm long with a radius of 4 cm (homogeneous flux).	Doxorubicin/Adriamycin (25 ng/ml)	K562 human myelogenous leukemia cell line	Decreased P-glycoprotein expression led to an increase in drug accumulation inside cells and reduced drug efflux. This synergy also induced DNA damage that led to cell cycle arrest at the G2/M phase, alongside alterations in cell ultrastructure (depressed membrane, protuberances, larger vacuoles). SMF alone did not induce detectable DNA damage, it markedly enhanced ADM-induced cytotoxicity and DNA lesions, likely by potentiating drug action rather than directly damaging DNA. ADM alone increased P-glycoprotein (P-gp) expression-contributing to multidrug resistance- the addition of SMF significantly suppressed ADM-induced P-gp expression, thereby promoting intracellular drug accumulation and reducing drug efflux.	Synergistic effect-Significant enhancement of Adriamycin cytotoxicity compared to drug alone.	^67^
9	24	Solenoid generated a homogeneous magnetic field with less than 1% field variation.	Paclitaxel/Taxol (Alkaloid) (10 ng/mL)	K562 human leukemia cell line	Increased cell membrane permeability that increases uptake of drug. SMF alters plasma membrane organization and fluidity, affecting ion transport, membrane permeability, and drug-membrane interactions; enhances drug uptake and potentiates membrane damage induced by chemotherapeutic agents.	Additive effect-Increased cell membrane holes and damage, but the overall effect was essentially the sum of SMF-induced and Taxol-induced effects.	^68^
9	12		Doxorubicin/Anthracycline(25 ng/mL)			Synergistic effect- Synergistic effects with doxorubicin producing larger and more severe membrane disruptions.	
9	12		Cisplatin (10 µg/mL)			Synergistic effect- Synergistic effects with cisplatin producing larger and more severe membrane disruptions.	
9	24		Cyclophosphamide (0.4 mg/mL)			Additive effect- Slightly increased hole size on the membrane, but the total membrane damage was only additive.	
10	24 & 48	Two large coils, 3 mm diameter and ~1 km length, powered by a direct current. The coils were positioned between 2 iron blades, between these blades there was a exposure unit, containing the samples with controlled temperature, humidity and CO_2_ (homogeneous flux).	Doxorubicin (0.1 μM)	MCF-7 human breast cancer cell line	Doxorubicin initiates redox cycling, producing superoxide (O_2_^.-^) which in turn facilitates the release of iron (Fe^2+^) from ferritin. This released Fe^2+^ and H_2_O_2_ participated in Fenton reaction to generate hydroxyl radicals (OH). Thus, combined effect results in decrease in intracellular iron concentration and increased ROS production by 2-fold. Modulation of ROS levels, inhibition of P-gp expression, disruption of cell membrane integrity, enhanced DNA damage, cell cycle arrest at G2/M.	Synergistic effect- Enhanced the cytotoxicity of doxorubicin, increased intracellular ROS, and induced greater cell membrane damage, resulting in synergistic killing effect.	^114^
10	24 and 48	Two large coils, 3 mm diameter and ~1 km length, powered by a direct current. The coils were positioned between 2 iron blades, between these blades there was a exposure unit, containing the samples with controlled temperature, humidity and CO_2_ (homogeneous flux).	Cisplatin (*cis*-diamminedichloroplatinum (II)) (IC_50_ 12 and 3 μg/mL)	HeLa human cervical cancer cell line	Increased G2/M phase arrest, necrosis and apoptosis. Co-treatment of HeLa cells with cisplatin and SMF for 48 h significantly increased G2/M cell cycle arrest, late apoptosis, and necrosis compared to cisplatin alone.	Synergistic effect-Enhanced cisplatin-induced apoptosis influenced cell cycle progression by modulating key regulators. Co-treatment of HeLa cells with 10 mT SMF and the IC50 dose of cisplatin for 48 h drastically reduced cell viability from 51% to 11%, indicating a strong synergistic cytotoxic effect.	^141^
15	24, 48, and 96	Two large coils, 3 mm diameter and ~1 km length, powered by a direct current. The coils were positioned between 2 iron blades, between these blades there was a exposure unit, containing the samples with controlled temperature, humidity and CO_2_ (homogeneous flux).	Cisplatin (μg/mL) A2780 (Sensitive): 27.69 ± 9.58 (24 h); 3.55 ± 3.14 (48 h); 1.72 ± 1.09(96 h) A2780-CP (Resistant): 61.15 ± 13.91 (24 h); 14.86 ± 4.8 (48 h); 6.19 ± 1.11(96 h)	A2780 human ovarian cancer cell line (sensitive)	In sensitive ovarian cells, increased *CTR1* expression enhanced cisplatin uptake, resulting in greater DNA damage and the upregulation of *P53* and *P21*, thus promoting apoptosis. In cisplatin-resistant ovarian cells, the response was weaker, with modest DNA damage and apoptosis, but a prominent increase in necrosis was observed.	Synergistic effect- Elevated ROS via altered intracellular iron homeostasis and enhanced hydroxyl radical formation, leading to lipid peroxidation and DNA damage; Upregulated apoptotic genes P53 and P21 and CTR1, a transporter enhancing cisplatin uptake, while anti-apoptotic Bcl2 remained unaffected.	^138^
				A2780-CP human ovarian cancer cell line (resistant)			
31.7–232	24	Three NdFeB magnetic parallelepipeds (50.8 ×50.8 ×25.4 mm), coated with nickel, grade N40, Br: 1260–1290 mT, magnetized across the thickness and contained in six shelves of plexiglass (inhomogeneous SMF).	Cisplatin (0.1 μM)	SH-SY5Y human neuroblastoma cell line	Decreased cisplatin toxicity, resulted in increased cell viability (~15%); decreased cleaved caspase-3 (by 80%), and reduced ROS production (by 18%), Co-exposure to SMF significantly attenuated cisplatin toxicity, increasing cell viability by 15% compared to cisplatin alone, while markedly reducing apoptosis and oxidative stress, as indicated by 80% lower cleaved caspase-3 levels and 18% reduction in ROS production.	Antagonistic effect- Exposure of cisplatin-treated cells (0.1 mM) to a SMF for 2 h significantly enhanced cytotoxicity, evidenced by a up to 30% reduction in cell viability, marked overexpression of cleaved caspase-3 (up to 46%), increased ROS production (up to 23%), and a decrease in mitochondrial content, SMF for up to 24 h did not affect SH-SY5Y cell viability, although it increased ROS levels and altered cell morphology. However, when combined with low-dose cisplatin, SMF significantly reduced cisplatin-induced cytotoxicity by preserving cell viability and suppressing ROS generation and caspase-3 activation, indicating (antagonistic) effect of SMF against cisplatin in these cells.	^143^
500	72	Cylindrical permanent (NdFeB)	Capsaicin (50 μM)	HepG2 human liver cancer cell line	Increased binding of capsaicin with the TRPV1 receptor led to an increased intracellular [Ca^2+^], elevated Bax/Bcl-2 ratio and enhanced caspase-3 activation, thereby induce apoptosis.	Synergistic effect-Significantly enhanced capsaicin-induced cytotoxicity, increased apoptosis, reduced cell viability compared to capsaicin alone. Treatment with 50 μM capsaicin for 72 h markedly increased apoptosis in HepG2 cells, raising apoptotic cells from 1.5% in controls to 32.1%. Co-exposure to a SMF further potentiated capsaicin-induced apoptosis to 59.3%, demonstrating a strong synergistic enhancement of anticancer efficacy by SMF.	^79^
700	24	The magnetic chamber consisted of a ferromagnetic yoke and four permanent neodymium magnets placed symmetrically on the top, bottom, left, and right (homogeneous SMF).	Baicalin or Baicalein (Flavones) (50 µmol/L)	C32 human amelanotic melanoma cell line	Decreased mRNA expression level of *SOD1, SOD2* and *GPX1* and normalized antioxidant enzymatic activity, thereby diminished redox-mediated cytotoxicity.	Antagonist effect-Treatment with baicalin or baicalein (50 µM) alone induced oxidative stress, increased antioxidant enzyme activity (SOD, GPx, CAT), and upregulated antioxidant genes (SOD1, SOD2, GPX1, CAT), consistent with their known anticancer, pro-oxidant, and apoptosis-inducing properties. Simultaneous exposure to a moderate-intensity SMF normalized antioxidant enzyme activity and reduced flavone-induced gene expression at the mRNA level. SMF alone did not disturb redox balance in melanoma cells.	^125^
700	24	Magnetic chambers with a ferromagnetic yoke and permanent magnets.	Caffeic Acid (CA) (1 mmol/L)	C32 human amelanotic melanoma cell line	Caspase pathway activation increased caspase 3 activity, upregulated pro-apoptotic *Bax* and downregulated anti-apoptotic *Bcl2* expression. Induction of oxidative stress, modulation of antioxidant enzyme activity, redox homeostasis modulation, activation of apoptosis pathways (ROS-mediated, mitochondrial-dependent apoptosis).	Synergistic effect-Caffeic acid alone induced apoptosis; Co-exposure with SMF altered redox balance and modulated apoptotic efficiency, indicating interaction between SMF and phenolic compound. Chlorogenic acid induced apoptotic effects; SMF modified the extent of apoptosis, suggesting a non-additive interaction dependent on oxidative stress regulation.	^144^
			Chlorogenic Acid (CGA) (1 mmol/L)				
1000	72	Permanent magnets	0.1 uM-0.5 uM 5-Fluorouracil, 0.1 uM 5-Fluorouracil + 1 nM Paclitaxel and 0.1 uM-0.5 uM Cisplatin	HeLa human cervical cancer, MCF-7 breast cancer, CNE-2Z nasopharyngeal carcinoma and HCT116 colorectal cancer cell line	SMF disrupted microtubules, causing abnormal spindle formation and mitotic arrest. SMF disrupts mitotic spindle formation, causing cell cycle arrest; enhances microtubule-targeting and DNA synthesis inhibition by 5-FU. SMF alters spindle orientation and microtubule dynamics; synergizes with Paclitaxel-induced mitotic arrest.	Synergistic effect-Combined treatment increased cytotoxicity and apoptosis compared to 5-FU alone. Combined treatment showed higher mitotic arrest, reduced proliferation, and enhanced apoptosis relative to Paclitaxel alone. Exposure to 1 T SMFs enhanced the anticancer effects of 5-FU and 5-FU + Taxol, but not cisplatin. This indicates that SMF’s combinational effects with chemotherapy are drug specific.	^88^
1000	72	Coaxial coils with an iron core in a Helmholtz configuration, using copper wire wound around a ring form (homogeneous).	Mixture of antineoplastic drugs (5-Fluorouracil, Cisplatin, Doxorubicin, Vincristine)	HL-60 human promyelocytic leukemia cell line	Enhanced cytotoxicity by reducing metabolic activity likely *via* the radical pair mechanism. SMF may alter radical lifetimes and biochemical reaction rates, leading to modulation of lipid peroxidation, calcium signaling, transcriptional activity, and apoptosis pathways.	Synergistic effect-Significant reduction in metabolic activity up to 81%. SMF exposure inhibited proliferation, induced differentiation/apoptosis-like responses.	^65^
**Radiation therapy**							
80	6 or 20 h (during recovery) and 6 h (before XR) + 6 or 20 h (during recovery)	Custom-made 4 × 4 cm Neodymium magnetic plaque was placed 1 mm beneath the cell monolayer generate axial magnetic field.	5 Gy X-ray (dose rate of 1.1 Gy/min)	Primary glioblastoma cells	Decreased the extent of X-ray-induced DNA fragmentation/damage and largely averted the loss of mitochondrial membrane potential. SMF modulates radiation-induced free radical lifetimes and DNA repair kinetics, affecting oxidative DNA damage formation.	Antagonist effect-SMF exposure significantly modulated X-ray-induced DNA damage in human primary glioblastoma cells, demonstrating that SMF can alter cellular radiosensitivity without being genotoxic on its own, likely through effects on free-radical dynamics and DNA damage/repair processes.	^235^
	0 to 24 (following irradiation)				No significant difference but significant increase in Tail DNA % at the 1st hour in case of iSMF2 group.	No significant effect but iSMF2 suggests a delay in DNA repair.	
1070 ± 0 0.037	24	North pole of permanent magnet	4–10 Gy X-ray (1.04 Gy/min)	MDA-MB-231 and MCF-7 human breast cancer cell lines	SMF acted as antioxidant, decreased the X-ray-induced ROS concentration. No change in X-ray induced cytotoxicity. X-ray–induced DNA damage and cell death occurs largely via ROS -independent mechanisms; reduction of intracellular ROS does not significantly alter radiation-induced cytotoxicity.	No effect-Despite a relatively small increase in ROS (<20%) in MCF-7 cells compared with MDA-MB-231 cells (40-90%), 4-10 Gy X-ray irradiation markedly reduced cell numbers and increased cell death in both cell lines. With SMF reduction of intracellular ROS did not attenuate X-ray–induced cytotoxicity, confirming that the anticancer effect of X-ray irradiation in breast cancer cells is predominantly mediated by ROS-independent DNA damage mechanisms.	^203^
1500	SMF exposure occurred simultaneously with radiation treatment. Biological effect of treatment was analyzed after 24 h (short term exposure) and 168 h (short term exposure) post treatment.	The radiation treatment setup combined the photon beam from a linear accelerator (Linac) and the magnetic field generated by an electromagnet. The Linac’s isocentre coincides with the center of the electromagnet poles, and the photon beam is shaped to cover the sample zone.	6 Gy of 6 MV X-Ray	PANC-1 and AsPC-1 human pancreatic cancer spheroids cell lines cultured in 3D polyurethane scaffolds	The study performed In Vitro hypoxia (1% O2) and In Vitro Normoxia (21% O2) condition. Increased apoptosis in hypoxic condition at both 24 and 168 h post treatment, and enhanced apoptosis in normoxic condition at 168 h long term time point. The SMF 1.5 T enhanced the biological impact of X‑ray irradiation by increasing radiation‑induced apoptosis (Caspase 3/7 activity) in pancreatic cancer cells within a 3D scaffold, likely via modulation of radiation response pathways, including enhanced free‑radical–mediated DNA damage and altered DNA damage response under both hypoxic and normoxic conditions.	Synergistic effect-1.5 T SMF with 6 Gy X‑ray irradiation significantly increased apoptotic cell death and reduced cell viability compared with radiation alone at both 1- and 7-days post‑treatment in hypoxic and normoxic conditions, demonstrating that SMF can enhance the antitumor efficacy of radiotherapy.	^236^
1500	0.25 h a day for up to 3 days (for UMSCC-47 cells) or up to 4 days (for other three cell lines)	MRL- Devices that combine MRI with linear accelerators MR-Linac (homogeneous flux).	Single exposure treatment: 2, 5 or 6 Gy of 6 MV X-ray Fractionated exposure treatment: 2 Gy of 6 MV X-ray for 1, 2, 3, or 4 days (total dose up to 8 Gy)	H460 and H1299 human non-small lung cancer cell line, and UMSCC-47 and HN5 human head and neck squamous cancer cell line	No measurable effect on the response to X-ray radiation of four human cancer cell lines. SMF generated by the MR‑Linac does not alter the cellular radio response mechanisms (such as DNA damage fixation or repair) when human solid tumor cells are irradiated with 6‑MV X‑rays, indicating that under these conditions the magnetic field does not influence ionizing radiation’s biological effectiveness.	No effect	^237^
**In Vivo studies**							
587	2 h on day 5, 7 and 9 after tumor implantation	A cylindrical NdFeB permanent magnet produced an axial magnetic field (homogeneous flux).	Paclitaxel (5 mg/kg) (1.5 h intravenous infusion during SMF exposure)	Syrian Golden hamster bearing syngeneic A-mel-3 melanomas in dorsal skinfold chamber	Increased tumor growth inhibition compared to either treatment alone. SMF increased tumor microvessel leakiness and reduced blood flow, which promoted drug uptake.	Synergistic effect-Enhanced paclitaxel delivery to tumor tissue, increased tumor growth inhibition, improved antitumoral efficacy compared to paclitaxel alone.	^69^
22,000 and 150	4 h/day for 6 consecutive days	Water-cooled magnet, which used circulating water to maintain its temperature. It included magnet cavity (50 mm diameter and 1300 mm length) for generating SMF. Mice were housed in non-magnetic stainless-steel tubes (38.5 mm; 80 mm length) placed inside a removable inner cylinder (41 mm diameter; 700 mm length). Air circulation (450 L/min) an lung cancer erd temperature control was maintained to ensure oxygen supply and thermal regulation.	Platycodin D (1 week after injection of A549 cells, 2 mg/kg drug was injected into mice at 1-day intervals for 2 weeks)	24 mice, 25‑day‑old (~16 g) male BALB/c (Nu/Nu) bearing lung cancer A549 cells subcutaneously	Downregulated expression of genes such as *CD36* (scavenger receptor involved in angiogenesis and inflammation), and *VIPR1* (a vasoactive intestinal peptide receptor linked to tumor growth), while also regulated the expression of *CACNG5* gene. This mechanistic change resulted in 3.6-fold higher antitumor effect compared to Platycodin D alone. SMF modulates tumor microenvironment and cellular stress responses; platycodin D induces apoptosis and inhibits proliferation; combined treatment enhances antitumor efficacy without increasing systemic toxicity.	Synergistic effect-Moderate (150 mT) and ultra-high (22 T) SMF significantly enhanced platycodin D–mediated tumor suppression while reducing systemic toxicity, demonstrating a safe and effective combinational strategy for lung cancer treatment.	^147^
9400	200 (10 h/day for 20 days)	SMF was produced by a vertical superconducting magnet with a 100 mm diameter room temperature bore to generate SMF, maintained at 22–24°C using a water controlled double layer non-magnetic cylinder (87 mm inner diameter). Mouse were housed in a stainless-steel exposure device with 81 mm diameter, which was placed inside the bore (homogeneous flux).	Imatinib mesylate (20 mg/kg)	Female BALB/c (nu/nu) mice bearing human gastrointestinal stromal GIST-T1 cells subcutaneously; (systemic toxicity and behavioral assessment)	Resulted in 92.75% tumor suppression compared to high dose imatinib mesylate (80 mg/kg), while markedly decreased the toxicity related to imatinib mesylate.	Synergistic effect-9.4 T SMF alone for 200 h inhibited tumor growth by 62.88%. Notably, combining 9.4 T SMF with a low dose of imatinib mesylate (20 mg/kg) achieved 92.75% tumor suppression, which is comparable to the antitumor efficacy of a high imatinib dose (80 mg/kg), indicating that SMF markedly enhances drug efficacy while allowing dose reduction. High-dose imatinib (80 mg/kg) induce marked toxicity and depressive behavior in mice, whereas 9.4 T SMF significantly reduced these adverse effects, particularly mitigated depression-like symptoms.	^213^

### Other emerging biochemical targets of SMF stimulation

Alongside other prominent biochemical targets mentioned above, SMF stimulation may influence other signaling pathways through processes discussed already and other additional broader systemic responses in cancerous cell lines (Tables 1–3). SMF stimulation has been implicated to alter broad (nonspecific) cellular parameters such as pH^115^, metabolic activity^65^ and cell size^139^ as well as the expression levels of specific genes/proteins regulation (such as that of the tumor suppressor p53)^37,131,138^. Reports also suggest an adverse impact on cell cycle progression, predominantly leading to G2/M cell cycle arrest^40,67,127,140,141^. These changes can be linked to changes of the gene expression (transcriptome alteration), having knock-on effects on cellular levels of anti/pro-apoptotic proteins or modulation of pathways like Akt/mTOR^60,142^ and caspase activation^79,138,140,143,144^. External to the cell,—in addition to the previously discussed reorientation of collagen fibrils, SMF stimulation also modulates extracellular matrix glycosaminoglycans synthesis^145^, ultimately alters cell adhesion and migration. The influence on cell adhesion is likely multifaceted, with researchers also proposing disruption of integrin receptor function, which is crucial for mediating cellular attachment to extracellular substrates and integrating extracellular signals^146^. Moreover, SMF stimulation also influences broader biological processes such as immune function^63,147^ and angiogenesis^147,148^, indicating their usefulness in potential efficacy when used alongside immunotherapy.

**Table 3:** The influence of SMF stimulation on tumor growth in mice at different intensities, exposure durations, magnetic field directions and magnet types.

Direction	Intensity (mT)	Duration of stimulation	Type of Magnet	Mice and Tumors	Tumor growth	Explanation provided	Reference
Axial component normal to skin surfaces/tumor plane	586	3 h per day (Repeated on days 1, 2, and 3 after tumor cell implantation; total 3 exposures)	Cylindrical permanent magnet derived from NdFeB (homogeneous flux)	Syrian Golden hamster (Male, 6–8 weeks old) bearing syngeneic A-mel-3 amelanotic melanomas in dorsal skinfold chamber	30% growth inhibition compared to controls by day 10 while significant difference observed from day 5 onwards	Inhibited angiogenesis and microcirculatory impairment which maybe explained mechanistically through interference with calcium signaling, antioxidant defense, or membrane lipid orientation.	^148^
Axial	587	Protocol A: 2 h on days 3, 6, and 9 Protocol B: 35 min daily from days 3–11 (Only Protocol A showed efficacy)	Cylindrical permanent magnet derived from NdFeB (homogeneous flux)	Male C57B1/6 mice (immunocompetent) and male CD-1 nu/nu (immunodeficient), both bearing LLC-1 (Lewis Lung Carcinoma) cells (EGFR-overexpressing) in the dorsal skinfold chamber	Protocol A: 46% reduction in tumor size. Protocol B: No significant inhibition. Combination (SMF + Cetuximab): No additive synergistic effect; SMF appeared to interfere with Cetuximab efficacy (which alone caused 53% reduction).	Disrupted EGFR signaling pathway. Also, SMF exposure plausibly interfered with the binding or efficacy of the targeted antibody (Cetuximab).	^61^
Upward: Field lines directed upwards through the tumor.; Downward: Field lines directed downwards. (Only Upward was effective)	50–500	6 h per day, for 38 days	Neodymium N38 permanent magnet (horizontally homogeneous flux)	Female SPF BALB/c (*nu/nu*) mice bearing human gastrointestinal stromal GIST-T1 cells	Direction-Dependent Inhibition - Upward SMF: Significant inhibition (19.3% reduction in tumor weight).;Downward SMF: No significant effect on tumor growth.	-	^202^
Upward (Field lines directed upwards through the samples/mice) Note: Superconducting magnet samples were placed in the “upper part” (0.5 T). Permanent magnet plates for mice also provided upward field.	500 (Range: 100–500 mT across the uneven surface of permanent magnets; controlled 500 mT in superconducting magnet)	In Vivo (Mice): Protocol A (Superconducting): 10 h/day, 7 days/week for 6 weeks (Total 420 h) Protocol B (Permanent Plate): 24 h/day, 7 days/week for 6 weeks (Total 1008 h)	1. Superconducting Magnet (9.4 T max, samples at 0.5 T position) 2. Permanent Magnet (Neodymium N38) - Custom plate (250 × 160 × 45 mm) for mice	Female BALB/c *(nu/nu*) mice (6 weeks) bearing SKOV3 cells administered intraperitoneally	-Metastasis: Significant reduction in number of metastatic nodules (esp. in liver/intestine). - Tumor Weight: No significant change in total weight, but reduced nodule count.	Increased ROS concentration	^117^
Upward	Mice: Resin plates with button magnets (Surface avg: ~0.18 T for “0.3 T” plate; ~0.31 T for “0.6 T” plate).	Continuous exposure (24/7) starting from 3 weeks of age (PyMT model) or during treatment period.	Resin fiber plate embedded with a neodymium permanent magnet (horizontally homogeneous flux)	- PyMT Transgenic Mice (Spontaneous mammary tumor) - C57BL/6 Mice (B16-F10 melanoma)	- PyMT Model: Significant delay in tumor onset and reduced tumor growth with 0.6 T plate (0.3 T was ineffective). - B16-F10 Model: Adoptive transfer of SMF-treated CTLs significantly inhibited tumor growth and extended survival compared to control CTLs. tumor-infiltrating CD8 T cells isolated from SMF-exposed mice produced significantly increased amounts of antitumor granules and cytokines (granzyme B, IFN-γ, and TNF-α) compared to control mice	Mitochondrial Respiration Enhancement - Mechanism: SMF upregulated mitochondrial respiratory chain genes Uqcrb (Complex III) and Ndufs6 (Complex I). - Magnetoreception: This upregulation was mediated by candidate magnetoreceptor genes Isca1 and Cry1/Cry2. - Metabolic Boost: Increased ATP production and mitochondrial respiration (OCR) fueled the enhanced effector function and cytotoxicity of CD8 + T cells.	^63^
Upward vs. Downward	9400 (Ultra-high field, center of superconducting magnet)	88 h (8 h/day, 11 times, every other day)	Superconducting magnet with a 100 mm diameter bore (homogeneous flux)	Male BALB/c (nu/nu) mice bearing A549 cells subcutaneously	-Upward: Significant 31.2% reduction in cell number; 44.7% inhibition of tumor growth In Vivo. -Downward: No significant reduction in cell number or tumor growth (despite inhibiting DNA synthesis).	ROS-P53-Mediated G2/M Arrest - DNA Synthesis: Both directions inhibited DNA synthesis (likely via Lorentz forces on charged DNA/Topoisomerase II). - Direction Specificity: Only Upward SMF significantly increased ROS levels and activated P53 (p-P53 S15). - Cell Cycle: Upward SMF caused G2/M arrest (increased p-CDK1, Cyclin B1) and decreased mitotic index, translating to effective growth inhibition. Downward SMF inhibited DNA synthesis but may have accelerated cell cycle entry (forced mitosis), canceling out the inhibitory effect.	^37^
Upward	9400	200 h (10 h/day for 20 days)	Superconducting magnet with a 100 mm diameter bore (homogeneous flux)	Female BALB/c (nu/nu) mice bearing human gastrointestinal stromal GIST-T1 cells subcutaneously	- SMF Alone: 62.88% tumor inhibition (TGI). - SMF + Low Dose Imatinib (20 mg/kg): 92.75% TGI (comparable to high dose 80 mg/kg Imatinib alone at 95.49%). SMF significantly alleviated Imatinib-induced side effects: liver/kidney toxicity, weight loss, anxiety, depression, and social deficits.	- Side Effect Relief: SMF improved mental health (anxiety/depression) possibly via modulation of neurotransmitters or oxytocin/CaMKII pathways (citing previous work). -Synergy: SMF enhanced the efficacy of low-dose drug to match high-dose levels while mitigating toxicity, potentially by altering membrane permeability or vascular normalization.	^213^

## The consequences of SMF stimulation for healthy cells

For SMF stimulation to succeed as a therapeutic paradigm, it must ablate tumors while causing negligible influence on noncancer cells. A large body of work suggests that SMF stimulation increases or decreases the cell viability, cell proliferation rates and/or their mechanical orientation in a variety of noncancer cell lines (Table 4). In this section, we present a systematic compilation detailing the different consequences of SMF stimulation at the tissue scale.

**Table 4:** The influence of SMF stimulation on non-cancerous cell function at different intensities and exposure durations.

Intensity (mT)	Duration of stimulation (h)	Type of Magnet	Cell type	Outcome of SMF stimulation	Reference
0.06–0.12	24 h (2–4 days)	Cells were placed vertically between a pair of Helmholtz coils mounted on a Plexiglass shelf inside a CO₂ incubator.	Human umbilical vein endothelial cells	Increased cell proliferation (60–120 mT, 2-3 days) upto 40% and improved endothelial functionality associated with upregulation of eNOS expression without altering VEGF expression or NO concentration.	^154^
0.16–0.2 (stimulatory) and 0.4 (inhibitory)	144, 168, and 192	Circular permanent ring magnet placed coaxially inside a magnetic shielding chamber, placed in incubator. Cells were positioned inside the chamber at controlled distance from the magnet.	Primary rat skeletal muscle satellite cells	Accelerated myoblast fusion and myotube formation and increased the frequency of spontaneous contractions via RyR-mediated Ca²⁺ release from the sarcoplasmic reticulum (0.1-0.2 mT). While depletion of sarcoplasmic reticulum Ca²⁺ stores and decreased contractile activity, particularly in immature myotubes (0.4 mT).	^239^
2	3 h every 6 h for 8 days or 5 h every 6 h for 8 days	Cells were placed in the center of a custom-built tubular solenoid (PVC tube with 450 Cu turns) inside a CO_2_ incubator	Primary rat chondrocyte cells	Increased cell proliferation (3 h every 6 h for 8 days) and increased in glycosaminoglycans synthesis (5 h every 6 h for 8 day).	^145^
2	0.002 (1 min)	A custom-built circular coil (100-wire, 4 cm diameter) was mounted on the confocal microscope stage and encircled the observation chamber, with cells positioned inside the chamber at the center of the magnetic field.	Porcine granulosa cells	Membrane depolarization with increased cytosolic [Ca²⁺] and decreased mitochondrial activity (reversible upon field removal).	^240^
2	24, 48, 72 and 96	Cells were positioned on a plexiglass platform at the center of the solenoid inside a humidified incubator.	Swine granulosa cells (ovarian cell)	Modified the actin and alpha-tubulin cytoskeleton; these changes in cytoskeleton attributes lead to changes in cell morphology, decreased proliferation, and decreased mitochondrial metabolism. Metabolic modulation, reflected by altered steroidogenesis and energy metabolism.	^241^
5	48	Six square neodymium–yttrium–iron permanent magnets were bonded to form larger rectangular magnets, arranged as north and south poles. The cells were positioned between the magnet poles.	Human umbilical artery smooth muscle cells	Inhibited cell proliferation (~16%), migration (~25%), and adhesion (~33%) by suppressing clustering of integrin β1, inactivationg of focal adhesion kinase (FAK) via decreased FAK phosphorylation and decreasing cytosolic [Ca^2+^].	^150^
5-20	24 and 48	Two large coils, 3 mm diameter and ~1 km length, powered by a direct current. The coils were positioned between 2 iron blades, between these blades there was a exposure unit, containing the flask with cells with controlled temperature, humidity and CO_2_.	Human foreskin fibroblast cells	Time and intensity-dependent decreasee of cell proliferation and viability, driven by SMF-induced ROS generation and disruption of iron redox homeostasis.	^114^
140	72	Cells in well plates placed directly above the NdFeB permanent magnets	Human umbilical cord derived mesenchymal stem cells	Increased proliferation mediated through T-tyle Ca^2+^ ion channel-dependent membrane depolarization, which activates MAPK signaling pathways (ERK and JNK) and increased expression of proliferation associated transcription factors (FOS and EGR1).	^155^
<60 (Gradient SMF, up to 10000 mT/cm)	0.25, 0.5 and 0.75	Sample was placed above the magnetouch apparatus (disc-shaped device producing inhomogeneous gradient SMF)	Human peripheral blood neutrophil cells	[ROS] showed a time-dependent response, decreasing at 0.25 h, remain unchanged at 0.5 h, and increasing at 0.75 h exposure. Inhomogeneous SMF modulate ROS production in a time- and pole-dependent manner. Short exposure (15 min) consistently reduced ROS levels, whereas longer exposure (45 min) significantly increased ROS only under south-pole orientation.	^242^
80	Continuous throughout the duration of experiment (up to 5 days)	Cells were positioned directly above the Neodymium magnetic plaque, with the north pole oriented upward toward the cells	Myogenic L6 cells	Promoted myogenic differentiation via actin cytoskeleton remodeling; parallel alignment of myoblasts; enhanced actin stress-fiber formation; increased cell fusion efficiency; and accelerated formation of hypertrophic multinucleated myotubes without altering proliferation.	^160^
21.6	Continuous exposure for various durations:- Growth Kinetics: 12 days- Cell Cycle: 18, 24, 30 h- Differentiation: 21 days	Permanent Samarium Cobalt Magnets (SmCo5 cylinders; 4 cm thick, 9.5 cm diameter)	hUC-MSCs Human Umbilical Cord-Derived Mesenchymal Stem Cells	Increased proliferation & stemness through upregulation of pluripotency markers.	^156^
200	1	Cells were placed on the isocentre of a sectorial magnetic resonance tomograph equipped with an ultracompact permanent magnet	Human skin fibroblast cells	Altered morphology including spindle-shaped cells, stiff cytoplasmic protrusions, irregular plasma membranes, and reduced cell–substrate adhesion (due to sialic acid–containing sugar residues (D-Gal(β1 → 3)-D-GalNAc and β-D-GalNAc); decreased DNA synthesis and mitogenic signaling (downregulated Diacylglycerol and Inositol phosphates).	^169^
200	24 h/day for 3–7 days	Cells were placed above the NdFeB magnet	Rat bone marrow mesenchymal stem cells	Increased cell proliferation; enhanced alkaline phosphatase activity (early marker of osteogenic differentiation); and upregulated osteogenesis-related proteins (Bone Morphogenetic Protein-2 (BMP-2) and Collagen type I (COL I)), leading to stronger matrix mineralization by day 7.	^157^
230-250	18 h	Twelve solid magnets were used, with six magnets placed on each of two separate steel plates. The cells are positioned between the two magnetized steel plates.	WI-38 embryonic lung fibroblast cells	Increased [ROS] by 37% (decreased cell attachment and subsequent inhibition of cell growth.	^153^
500	168	Cells in well plates placed directly above NdFeB disc magnets, embedded well-to well beneath culture wells	Adipose-derived stem cells	Inhibited cell proliferation and viability; inhibited surface antigen expression (downregulated the expression of CD49d, CD54, and CD73); inhibited cytokine secretion (downregulated expression of VEGF, Insulin-like Growth Factor-1 (IGF-1), Transforming Growth Factor Beta 1 (TGF-*β*1)); inhibited stem cell genetic marker expression including Antigen-1 (Sca1), Octamer-4 (Oct-4), ATP-binding Cassette Subfamily B Member 1 (ABCB1).	^149^
500	4–8	Frozen cell suspension, placed in a double Dewar flask system, was positioned at the center of a standard iron-core electromagnet’s 4-inch pole gap	Mouse lung fibroblast L-929 and human fetal lung fibroblast WI-38 cells	Inhibited cell growth rate, loss of contact inhibition, and stable morphological and physiological transformation in L-929 cells (enlarged nucleoli, coarse chromatin forming intranuclear networks, and cytoplasm filled with dense perinuclear granules) while in WI-38 cells (extremely elongated cytoplasm, strongly basophilic nuclei, altered nuclear shape and presence of giant nuclei).	^151^
500	168	Cells were placed in the gap between the permanent neodymium magnets	Human adipose-derived mesenchymal stromal stem cells	Increased cell viability and proliferation potentially mediated by upregulation of αV and β3 integrins and activation of the PI3K/Akt signaling pathway; decreased apoptosis (downregulation of pro-apoptotic markers namely p53, p21, Bax and upregulation of anti-apoptotic marker Bcl-2).	^158^
500	120-168	Cells were positioned at th midpoint between two circular magnets placed opposite to each other.	Mouse neural progenitor cells	Increased proliferation and viability associated with upregulation of cell cycle regulatory proteins Cyclin B and increased Sox2 expression.	^159^
~1240 (Gradient SMF, up to 10^4 ^T/m)	48	Pair of axially magnetized NdFeB flat planar magnets arranged mainly in an antiparallel (NS–SN) configuration. Cells were cultured on glass slides positioned 0.1 mm above the magnets.	Naive (M0) peritoneal macrophages from C57BL/6 mice	Macrophages undergo extreme elongation; aligned according to the magnetic force (field-gradient) distribution; actin cytoskeletal remodeling; focal adhesion and Golgi complex disruption; TRPM2 channel clustering; polarization towards an anti-inflammatory M2 phenotype (upregulation of Arg-1 (M2 marker)).	^167^
3000	1	MRI system, cells positioned directly inside the MRI bore	Primary human chondrocytes	Decreased cell growth; increased apoptosis; transiently increased [Ca^2+^]; DNA fragmentation; upregulation of p53, p21, p27, and Bax protein; Increased phosphorylated ERK1/2.	^152^
4750	1	NMR Apparatus (NMRF)	Normal human peripheral blood mononuclear cells (PBMC) and Jurkat T-lymphoma cells	NMRF exposure caused no changes in proliferation, cytokine release, or Ca²⁺ homeostasis in PBMC, showing no adverse effects on normal immune cells whereas in Jurkat cell proliferation, accompanied by reduced IL-2 secretion and decreased intracellular Ca²⁺ levels, indicating selective disruption of calcium-dependent signaling in tumor cells.	^214^
8000	60	Cells were positioned at the center of horizontal superconducting magnet	Schwann cells from dissected sciatic nerves of neonatal Wistar rats	Schwann cells oriented parallel to SMF; actin fibers aligned with field direction, mediated by the Rho-associated kinase pathway.	^243^
8000	60	Horizontal-type superconducting magnet of 700 mm long with a 100 mm diameter bore.	Human kidney HEK293 cell line	Induced cell reorientation and realignment in a cell type- and density-dependent manner; effects are attributed to diamagnetic anisotropy of cellular components and may influence cell organization without directly causing cytotoxicity.	^168^
159 mT± 13.4 (homogeneous SMF) with 4 Gy 60Co-ϒ- irradiation (dose rate of 0.35 Gy/min)	24 (before irradiation)	Homogeneous SMF exposure chamber consisted of two ferrite block magnets (100 mm × 140 mm × 25 mm, Br = 400 mT) that faced each other with opposite polarity, thereby creating a uniform magnetic field. The active volume (140 mm × 100 mm × 50 mm) housed three vertically stacked petri dishes.	Human leukocytes	No significant difference in immediate DNA damage.	^205^
	0 to 24 (following irradiation)			Decreased tail DNA % at 4th hour. Antagonist effect (slight increased DNA repair).	
47,700 mT/m (iSMF1), 1200 mT/m (iSMF2) and 300 mT/m (iSMF3) (inhomo geneous SMF)	24 (before irradiation)	Inhomogeneous SMF exposure chamber consisted of cylindrical NdFeB N50 magnets (10 mm × 10 mm, Br = 1.47 T) that were arranged with a 10 mm lateral periodicity and alternating polarity. Magnets that faced each other in the matrices were also oriented with opposite polarity, generating an inhomogeneous field. The active volume (140 mm × 100 mm × 50 mm) housed three stacked petri dishes with the flux density and gradient vary across the petri dishes.		No significant difference in immediate DNA damage but a statistically significant difference was found between iSMF2 and iSMF3 group.	
	0 to 24 (following irradiation)			No significant difference but significant increase in Tail DNA % at the 1st hour in case of iSMF2 group.	

### Decreasing noncancer cell viability and proliferation rates

Several works indicate that SMF stimulation inhibits noncancer cell proliferation (Table 4)^114,149,150^. For example, in isolated human umbilical artery smooth muscle cells, 5 mT SMF stimulation for 48 h decreased cell proliferation, migration and adhesion by suppressing integrin β1 clustering, inactivation of Focal Adhesion Kinase (FAK) phosphorylation via decreased FAK phosphorylation, and decreasing cytosolic [Ca^2+^], which is critical for cell cycle progression^150^. In human foreskin fibroblast and MCF-7 cell lines, 5–20 mT SMF stimulation over 24 or 48 h decreased cell viability and proliferation by decreasing intracellular [Fe^2+^] and increasing intracellular ROS production, possibly through the radical-pair mechanism^114^. In adipose-derived stem cells obtained from inbred male Lewis rats, 500 mT SMF stimulation for 168 h decreased cell proliferation. This inhibitory effect was associated with decreased expression of surface antigen (CD49d, CD54, and CD73), downregulation of cytokines (VEGF, IGF-1, and TGF-β1), and decreased expression of stem cell markers (Oct-4, Sca-1, and ABCB1), without inducing detectable DNA damage^149^.

Apart from decreasing cell viability and proliferation, SMF stimulation has also been shown to decrease growth in non-cancerous cells (Table 4)^151–153^. In isolated human chondrocytes, 3000 mT SMF stimulation for 1 h decreased cell growth and induce apoptosis caused by transient increase in intracellular [Ca^2+^] and activation of apoptosis related signaling pathways (p53/p21/Bax), together with increased phosphorylation of extracellular signal regulated protein kinase (ERK1/2)^152^. Similarly, in heteroploid L-929 and diploid W-38 cells, 500 mT SMF stimulation for 4-8 h inhibited cell growth, resulted in loss of contact inhibition, and induced morphological and physiological alterations in cell^151^. In WI-38 embryonic lung fibroblast cells, 230–250 mT SMF stimulation for 18 h decreased cell attachment and subsequenct cell growth. These effects were associated with a transient increase in ROS production^153^. Collectively, these findings indicate that SMF stimulation across a range of field intensities and exposure duractions can inhibit noncancer cell growth and proliferation through mechanisms involving apoptosis signaling, oxidative stress, and alterations in cellular regulatory pathways, similar to mechanisms reported in tumor growth and cancer cell proliferation inhibition (Tables 1 and 3).

### Increasing noncancer cell proliferation rates

Although SMF stimulation clearly decreases the viability and proliferation rates of both noncancer and tumor cells, it also appears to exhibit paradoxical effects in some experiments, increasing noncancer cell proliferation rates (Table 4). For instance, in human umbilical vein endothelial cells, continuous 0.06 and 0.12 mT SMF stimulation for 48 hours increased the cell proliferation by 40% and endothelial fnctionality by upregulating the expression of endothelial nitric oxide synthase^154^. Similarly, in human umbilical cord-derived mesenchymal stem cells, 140 mT SMF stimulation for 72 h increased cell proliferation by regulating the opening of the T-type Ca^2+^ channels in the cell membrane, activating the MAPK cell signaling cascade and increasing expression of proliferation associated transcription factors FOS (Fos Proto-Oncogene, AP-1 Transcription Factor Subunit) and EGR1 (Early Growth response 1)^155^. Supporting these findings, another study on human umbilical cord-derived mesenchymal stem cells revealed upregulation of key pluripotency genes, thus enhancing stemness alongside accelerating cell proliferation^156^. Furthermore, in Sprague-Dawley rat bone marrow mesenchymal stem cells, 200 mT continuous SMF stimulation for 72 to 168 h increased cell proliferation, also resulted in increased alkaline phosphatase activity and increased expression of osteogenesis-related proteins, resulting in increased mineralization^157^. In human adipose-derived mesenchymal stromal stem cells, 500 mT SMF stimulation for 168 h increased cell proliferation by activating the phosphoinositide-3 kinase/Akt signaling pathway^158^. Extending these findings to wild-type neonatal mouse neural progenitor cells, 500 mT SMF stimulation for up to a 120–168 h resulted in increased cell proliferation by upregulation of cyclin B and increased expression of Sox2^159^. Together, these findings highlight that SMF effects on noncancer cells are context dependnet and can increase proliferation under specific fied strength and exposure conditions.

### Altering cell orientation and surface morphology

Beyond influencing cell viability and proliferation, SMFs of sufficiently high intensities (>1000 mT) can reorient healthy cells. As described in Section “The biochemical targets of SMFs", SMF stimulation alters intracellular [Ca^2+^], which is intricately connected to cytoskeletal reorganization (because Ca^2+^ binding depolymerizes microtubules and activates the functioning of actin-associated proteins). Altered cytoskeletal organization inside the cell can trigger changes in collagen organization outside the cell through integrins and focal adhesion proteins. Changes in cytoskeletal and collagen fibril organization can then work together to change cell orientation through mechanical cues. Indeed, SMF-induced cellular reorientation parallel to magnetic field lines has been recorded for diverse cell types, including myoblasts^160^, osteoblasts^161^, erythrocytes^162,163^, platelet cells^162^, mesenchymal stem cells^164^, Schwann cells^165^, smooth muscle cells^166^ and peritoneal macrophages^167^.

Extending these mechanistic and cell type observation, an intereting study demonstrate that SMF-induced cellular orientation is further influenced by cell morphology, proliferative state, and microenvironmental context. SMF stimulation of 800 mT for 60 h aligned spindle shaped human glioma GI-1 cells parallel to its direction. In contrast, human kidney HEK293 cell line (polygonal shaped) did not exhibit such alignment, representing shape dependent susceptibility to SMF. In addition to morphology, proliferative status and cell density also play critical roles in determining SMF reponsiveness. Notably, the orientation effect was observed only in actively dividing, high density human glioma GI-1 (canerous) cell line (cancerous) and also in rat vascular smooth muscle A7r5 (non-cancerous) cell line, only when the cells were actively proliferating at high cell density, indicating that cell density and cell divisions are critical factors in governing SMF-induced orientation^168^. These findings indicate that SMF-induced orientation is governed by an integration combination of cytoskeletal dynamics, cell shape and proliferative microenvironmental cues. This differential response will be of immense consequence while designing a selective SMF-based tumor therapy to understand the ramificantions for labile cell systems with stem or progenitor populations located in protected microenvironments (bone marrow niches, crypt bases, epidermal basal layers, hair follicle bulbs).

SMF stimulation also causes surface modifications in non-cancer cells. For example, in human lymphocytes, 6 mT SMF stimulation for 24 h resulted in change in glycoprotein distribution on the cell membrane and led to the appearance of surface protrusions^89^. Similarly, in human skin fibroblast, 200 mT SMF stimulation for 1 h induced significant plasma membrane alterations, including decreased exprssion of surface glycoconjugate sugar residues. These membrane compositions change were accompanied by spindle shaped morphology, irregular plasma membrane structure, and thin cytoplasmic protrusion^169^ (Table 4). These findings highlight that SMf exposure can alter plasma membrane composition and morphology. 

### Macroscopic changes due to SMF stimulation

Developing a detailed safety profile for SMF stimulation over the course of treatment is important for its application in a tumor treatment device. MRIs using intensities ranging from 64 mT through 7000 mT have been cleared by the USA FDA via the 510(k) premarket notification pathway^170,171^. However, these intensities should not be extrapolated to SMF stimulation because the time-invariance of the magnetic field would elicit different biomolecular responses as compared to those observed in MRI stimulation^172^. Notwithstanding, MRI regimen does involve an SMF component and thus, studies have been conducted to explore the effects of SMF to validate MRI treatments’ safety. A wide array of effects relating to physiological parameters has been observed in different subjects when exposed to various intensities. Cardiovascular haemodynamics, local and microvascular blood flow, neural function, autonomic nervous activity, pain perception are some of the parameters which are the metrics that have been considered in previous studies to assess the safety profile of SMFs. Contradictory findings in this regard have been enumerated in the discussion below by dividing the investigations based upon the intensity of SMF employed: Medium intensity SMF(<1000 mT) and High Intensity SMF (>1000 mT).

Medium intensity SMF: Investigation by Okano and Ohkubo found that exposure to 1 mT SMF for 30 min modulated pharmacologically induced vasodilation and vasoconstriction in the microcirculation of rabbits^173^. Likewise, 11 mT SMF stimulation for 200–250 s could inhibit action potential generation in cultured mouse sensory cells^174^. Additionally, another report confirmed reduced skin blood flow in human fingers owing to 400 mT SMF exposure for 15 min^175^. Another report suggested that in patients with diabetic neuropathy, it was only after 2 months of daily exposure to 45 mT static magnets that analgesic benefits became apparent with maximal changes observed during 3rd and 4th months^176^. This highlights the importance of long-term studies and indicates that even longer exposure studies are necessary for overall safety assessment. In regard to nerve function, in human exposure to either 1000 mT (for 1 h) or 45 mT (for over 4 months), SMFs did not alter nerve conduction velocities^176,177^. Also, it has been reported that there is an increase in the excitability of motor nerves when exposed to 1000 mT for 15 s (in humans) and 500 mT for 30 s (in rats)^178,179^. Moreover, studies done on humans concluded that magnets possessing strengths less than 1000 mT (up to 30 mins exposure) were incapable of causing any changes in muscle and skin blood flow at rest^180,181^ and during deep inspiration^182^. Along similar lines, a human study using mattress-embedded magnets (60 mT) showed no alteration in pain perception or hemodynamic responses during noxious stimuli, raising doubts over its benefits, as purported by the market^183^.

High Intensity SMFs: Even SMFs in the range of 1200- to 2000 mT could not alter nerve conduction velocity, membrane potentials, transmembrane currents, action potential amplitudes, and refractory periods^184–186^. However, exposure to even higher intensity 8000 mT SMF for 20 min did lower both temperature and skin blood flow in humans, though such effects were transient^187^. Studies on mice and rats indicate that 9400 mT SMF stimulation for several weeks does not cause physiological or cognitive damage^188–190^. Rigorous studies on human subjects affirm the overall safety profile of SMF applications in a similar range^191^, showing negligible cognitive or physiological damage (for parameters including blood pressure, oxygen saturation, heart rate, and body temperature) on SMF stimulation of up to 8000 mT for up to 1 h^192,193^. The only clinical parameter that was significantly altered appeared to be systolic blood pressure, which increased by 4% on 8000 mT SMF stimulation. The most frequently reported comment from subjects related to dizziness, but all effects were transient. Another study compared the influence of 8000 mT SMF stimulation through an MRI system on healthy adults for 1 h^193^. No significant changes were observed in either vitals (body temperature, blood pressure, heart rate, and respiratory rate) or cognitive functions measured by mini-mental status examination (MMSE)^194^, literal and semantic fluency tests^195^, and dexterity tests^196^.

Collectively, these studies demonstrate that further investigation is required to assess the safety profile of SMFs, both for short and long-term duration. However, we expect sustained stimulation over significantly larger timescales to be used for tumor treatment (on a days-to-weeks’ timescale, at least). Thus, performing studies understanding the physiological and cognitive consequences of SMF stimulation with different spatial profiles, over a wide range of exposure times, will be important for future work.

## Unraveling the discrepancies in SMF-induced tumor response

The previous sections have reviewed the mechanisms by which SMF stimulation decreases tumor health. However, some evidence also suggests that SMF stimulation can also have the opposite effect: that of enhancing tumor growth or only influencing it in a negligible manner. For example, in human neuroblastoma SH-SY5Y cell lines, a hypomagnetic 2 × 10^−4^ mT SMF stimulation for 48 h (after shielding out the geomagnetic field) increased cell proliferation by promoting G1-phase progression in the cell cycle, thereby accelerating cell division^197^. In the same cell lines, a separate study showed that $< 2\times {10}^{-3}{\rm{mT}}$ SMF stimulation for 48 h increased cell proliferation by decreasing ROS accumulation, possibly by altering the activity of superoxide dismutases, which reduce superoxide radicals into hydrogen peroxide^124^. In mouse 4T1 breast cancer cell lines, higher intensity (150 mT) SMF stimulation for 48–72 h increased proliferation by upregulating the expression of the e2f1 transcription factor, a key regulator of cell cycle^136^. Taken together, such findings imply that SMF stimulation, at least in the low intensity (<1000 mT) limit, can also initiate pro-growth signaling within tumors.

Thus, SMF stimulation can result in often contradictory biological responses contingent at least upon intensity, exposure duration, and cell type. Discrepancies in results also arise due to incompletely accounted for physiological and physical parameters used in experiments, which influence the intracellular response to SMF stimulation. In this section, we examine these parameters and demonstrate how seemingly minor experimental variations in these parameters can profoundly influence tumor biology and therapeutic outcomes. We also discuss the impediments to reproducibility of experiments that ultimately lead to variability and translate to discrepancies during meta-analyses.

### The physiological parameters that govern tumor response to SMF stimulation

The responses of cells to SMF stimulation exhibit significant variation across different cell types and are governed by cell type-specific architectural and molecular organization, including membrane composition and organization, cytoskeletal topology, organelle distribution and dynamics, and the abundance of anisotropic molecular constituents^60,198–201^. Different cell types can exhibit variation in size, shape and orientation (relative to the external SMF), as well as differences in the volume and spatial organization of diamagnetic, anisotropic components (e.g., membranes and cytoskeleton structures), resulting in different net magnetic susceptibilities of these cells, and leading to cell type dependent magnitudes of diamagnetic torque under SMFs. The shape of a cell determines its overall anisotropy in magnetic susceptibility, which dictates the extent to which it experiences an SMF-induced rotation. Additionally, cell shape is also key in determining the placement of various anisotropic cellular components, and thus, determining how the force vectors appear in the overall dynamics of the biological system. Because different SMF intensity profiles can elicit distinct intracellular responses, cell shape can play a crucial role in determining how tissues respond to SMFs. Shape-induced anisotropy may also explain the differential sensitivity of adherent cells (asymmetric shape) and leukemia cells (spherical/floating) to SMF stimulation in different directions (Table 3)^202^. Moreover, cell shape is dependent on the type of subject cells and thus, cell line under consideration is a crucial determinant of tumor response. Different cell lines derived from specific cell types of specific organisms are used in In Vitro experiments involving SMF stimulation, resulting in a diversity of responses^202^. For instance, exposure to 1000 mT for 24–48 h decreases ROS concentration in Human glioblastoma U251, human colon epithelial carcinoma HCT116, human hepatocellular carcinoma HepG2, human gastrointestinal stromal GIST-T1, human nasopharyngeal CNE-2Z, human bladder EJ1, human breast cancer MCF-7 and MDA-MB-231 cell lines while no change in ROS concentration has been observed in human prostate adenocarcinoma PC3, human cervical cancer HeLa, and CHO Chinese hamster ovary cell lines (Table 1)^203^, illustrating the variability in oxidative stress responses among different cell populations upon SMF stimulation. Building upon these cell type-specific oxidative differences, proliferative responses to SMF stimulation also exhibit similar heterogeneity (Table 1)^60,153^. When seven human malignant cell lines—representing diverse cancer origins (CNE-2Z, HCT116, A431, A549, MCF-7, PC3, and EJ1) were exposed to 1000 mT SMF for 48 h, varying effects were observed based on cell density and cell type. At high cell density, all cell lines except EJ1 exhibited proliferation inhibition. This ability of SMF to inhibit most cancer cells at high densities suggests their potential in treating solid tumors. Conversely, CNE-2Z, MCF-7, and EJ1 showed increased cell proliferation at low cell density, while the other cell lines (HCT116, A431, A549, PC3) showed no significant change. Additionally, exposure to 8000 mT SMFs for 60 h aligned spindle-shaped human glioma GI-1 cells parallel to the SMF direction. In contrast, human kidney HEK293 cell line (polygonal-shaped) did not exhibit such alignment, representing shape-dependent susceptibility to SMF exposure. Notably, the orientation effect was observed only in actively dividing, high-density human glioma GI-1 cell line, indicating that cell density and cell division may be key factors in governing SMF-induced orientation^168^. High Cell density (in spindle shaped cells) has been postulated to aid contact guidance, aligning the cells along the same long axis, and thus amplifying the torque generated by individual cells. These findings collectively demonstrate that SMF therapeutic efficacy is governed by complex interactions between cell type specific characteristics and cellular morphometric properties, particularly density-dependent factors.

### Magnetic field parameters that govern tumor response to SMF stimulation

The magnetic field parameters that define the treatment regimen, such as intensity, direction, and stimulation duration, dictate the downstream physiological responses to it. In this section, we discuss how cells respond to different magnetic field parameters, illustrating the inapplicability of a ‘one size fits all’ mechanism.

#### Magnetic field homogeneity and positioning of the biological sample relative to the magnet

The spatial geometry of the generated magnetic field, alongside the relative positioning of the biological target system, greatly influences the strength, uniformity and direction of the magnetic field experienced by the cells. Hence, the positioning of the target biological sample is itself contingent upon the magnetic field profile of the exposure system under consideration. Homogeneity of the magnetic field is of immense consequence since in an inhomogeneous field profile, the magnetic field intensity experienced by various components would be a function of their location in space, and hence introducing huge variations as regards the force/torque acting on them. Also, field gradients impart magnetophoretic forces on diamagnetic or paramagnetic entities (including whole cells, organelles, paramagnetic free radicals, andiron-rich vesicles), hence drastically affecting the dynamics of the biological system^204^. Due to this spatial variability, heterogeneous fields are difficult to reproduce using different SMF configurations. Notwithstanding, both homogenous and heterogenous fields may have potential therapeutic implications and hence must be studied well for translational research.

**Fig. 4:**
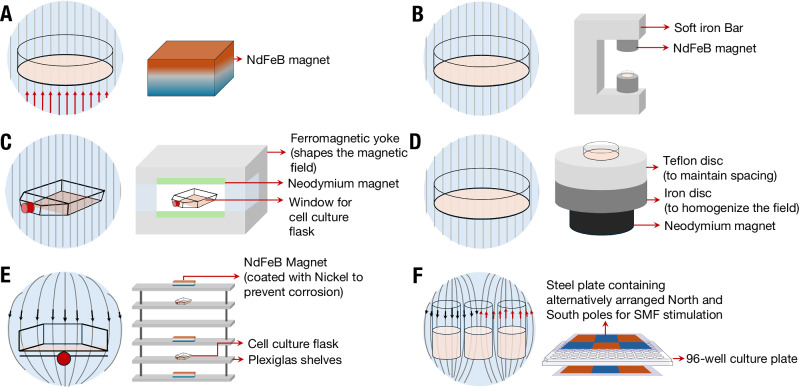
Schematic representation of spatiotemporal profile generated by permanent magnets in several experimental setups. A magnified illustration of the direction of magnetic field lines of force is shown on the right side of each figure (in blue). In all magnets, red represents the north pole of the magnetic and blue represents its south pole. The direction of magnetic field lines are illustrated using gray lines in zoomed-in schematics. The arrows of magnetic fields lines indicate their direction. Note that the number of field lines in a figure do not scale with magnetic field intensity. **A** Block magnet with culture plate positioned at its top. Created using details described in refs. ^69,79,112,202^. **B** C-shaped SMF exposure system constructed from soft iron bars with NdFeB pole magnets forming a air gap. Created using details described in ref. ^139^. **C** Magnetic chamber equipped with a ferromagnetic yoke and permanent neodymium magnets placed symmetrically on walls across culture flask. Created using details described in ref. ^125^. **D** Neodymium disk magnets placed beneath Petri dishes, with an interposed iron disc to homogenize the field and a Teflon disc to maintain spacing. Created using details described in refs. ^77,90^. **E** Three Ni-coated NdFeB parallelepiped magnets held in a plexiglass shelf structure with PVC bolts. Created using details described in ref. ^143^. **F** Twelve alternatively arranged rectangular magnets fixed with epoxy to two steel plates, with six magnets per plate. Created using details described in ref. ^153^.

Permanent magnets are frequently employed in SMF-based studies owing to their null power requirements as regards magnetic field generation. However, the field is generated through the inherent magnetization of ferromagnetic materials and is hence unchangeable. In the case of permanent magnets, this field profile is a function of the magnet’s shape. Different magnet shapes, including rectangular^136,202^, square^202^, cylindrical^69,79^, disc-shaped^112^, and C-shaped magnets^139^, have been employed in studies, each generating different magnetic profiles. For rectangular, square, cylindrical, and disc-shaped magnets, the culture plate is placed above the magnet when a homogenous field is required^69,79,112,136,202^. This is so because in the proximity of the magnet’s face the sample experiences near homogenous filed with minimal fringe effects (Fig. 4A). While for a C-shaped magnet, the sample is placed within the poles for a uniform magnetic field (Fig. 4B)^139^. In the case of a cylindrical magnet, a more symmetric field is created around the central axis because magnetic field lines flow from the north to the south pole symmetrically along the curved surface^69,79^. Like cylindrical magnets, disc magnets also produce strong surface fields, but the depth is limited^112^. Certain experimental setups even employ a ferromagnetic yoke (Fig. 4C^125,144^) or an iron disc (Fig. 4D^77,90^) to shape and homogenize the magnetic field for good measure. The arrangement shown in Fig. 4C^125,144^ exploits the fact that when two large magnetic blocks are placed opposite to each other, such that the gap between them is significantly smaller than the face width, we can assume field lines as perfectly uniform in the intervening gap. This can be described as an idealized “infinite sheet” approximation generating a homogenous field. Innovative configurations have also been adopted for producing heterogeneous SMFs —4E^143^ and Fig. 4F^153^ representing two such unique configurations. While in Fig. 4E the sample is interposed between only 2 magnets, in Fig. 4F, due to the placement of multiple magnets in a fashion of alternating polarity, there is a greater degree of inhomogeneity in the direction of magnetic field being experienced by the cells. The consequential nature of this kind of direction reversal is emphasized in the section of this work describing magnetic field direction. The setup shown in Fig. 4F involves wells arranged side by side (usual 96 well culture plate), while a configuration involving culture plates stacked vertically on top of one another has also been used previously^205^ showcasing use of diverse arrangements of the target cell populations to modify the field experienced by the cells.

Another way of generating SMF is through electromagnets, which are more tunable since their magnetic field intensity can be modified by adjusting the current supplied and the number of turns involved. However, electromagnets do require external power, and their working involves joule heating, making it difficult to maintain local temperature.

**Fig. 5:**
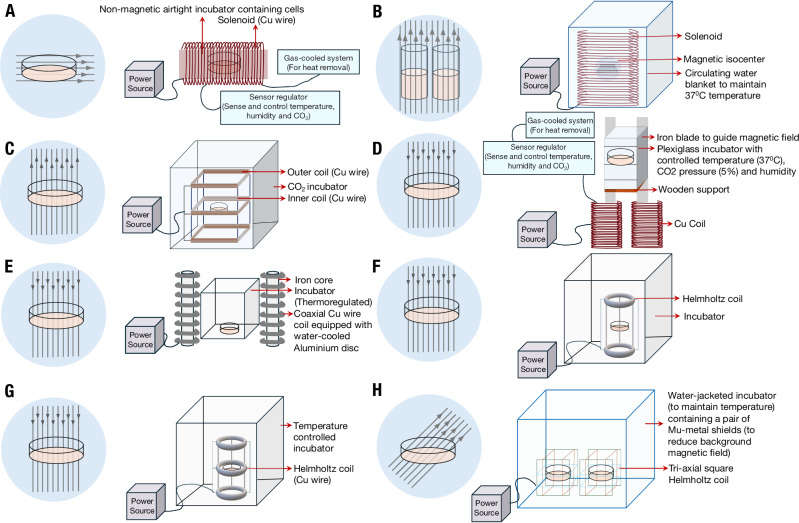
Schematic representation of spatiotemporal profile generated by permanent magnets in experiments. **A** Solenoid-based setup with enclosed incubator. Created using details described in refs. ^76,206^. **B** Solenoids forming a highly uniform isocentre. Created using details described in ref. ^22^. **C** Merritt-like coil setup inside incubator. Created using details described in ref. ^207^. **D** Coil system with enclosed incubator. Created using details described in refs. ^138,141,244^. **E** Coaxial coil with an iron core. Created using details described in refs. ^53,54^. **F** Two Helmholtz coils within incubator. Created using details described in refs.^115,140,207^. **G** Three Helmholtz coils within incubator. Created using details described in ref. ^140^. **H** Water-jacketed incubator housed two sets of tri-axial square Helmholtz coils and Mu-metal shields to reduce background magnetic fields. Created using details described in ref. ^115^.

Figure 5A–H represents diverse setups to ensure uniformity of the field using electromagnets. Figure 5A^76,206^ and Fig. 5B^22^ capitalize on the isocentre of the field profile generated while setup in Fig. 5C^207^ employs Merritt-like coil configuration installed within CO₂ incubators. This Merritt-like coil configuration yields a highly uniform field over a much larger volume as compared to a similarly sized solenoid’s central zone. Dual coil systems have also been ingeniously engineered to generate homogenous fields (Fig. 5D^114,138,141^ and 5E^53,54^). Notably, setup shown in Fig.5D uses a rectified AC power source with a ~ 5% ripple, which may lead to minor spatial heterogeneity and, hence, extra caution is required. We must pay attention to these minor details, as they could potentially introduce artefacts into the results and impede accurate interpretation, for they may induce artefacts in results and hinder accurate interpretation. In contrast, the setup depicted in Fig. 5E uses an DC power source, which is better suited for field integrity. Another interesting configuration involving multiple coils is a Helmholtz coil system with identical circular coils placed symmetrically along a common axis, as depicted in Fig. 5F^64^ and 5G^140^. Among the two-coil Helmholtz systems (Fig. 5F) and the three-coil Helmholtz systems (Fig. 5G), three-coil systems provide more uniformity and greater volume exhibiting uniformity, but that comes at the cost of ease of build. These Helmholtz systems aim to generate a volume with a uniform magnetic field, albeit smaller than that created in the case of Merritt coil configuration of similar dimensions. Also, the field uniformity takes a small hit in the case of Helmholtz coils when compared to the Merritt coil configuration. Notably, computer simulations using software like CST STUDIO can be immensely useful for ensuring homogeneity of field intensity within the exposure units, and hence their usage must be encouraged. However, Collectively, the choice of a magnetic field exposure system involves a series of engineering trade-offs balancing a lot of characteristics as discussed above.

Maintaining field homogeneity is further challenged when transferring In Vitro results into systems to In Vivo models, which uniquely pose several challenges. Both animal and human subjects require special arrangements to maintain field integrity while accommodating physiological constraints. The difference between the susceptibilities of tissues is relatively minimal as compared to tissue-air interfaces. At these tissue-air interfaces, the large difference in magnetic susceptibility between air and tissue causes the magnetic field lines to bend and concentrate (distort), creating “hot spots” (stronger field) and “cold spots” (weaker field). The same principle can be extrapolated while exposing lab animals like mice to SMFs. If ferromagnetic materials (like steel plates) are used in the setup to hold magnets, they create sharp, unpredictable distortions in the magnetic field right at the tissue’s interface. Thus, the tissue-magnet interface poses certain unique challenges when dealing with In Vivo setups due to possible field distortions. For experiments with mice, SMF exposure is typically achieved using custom designed permanent magnet assemblies, such as magnetic plates composed of neodymium cubes placed inside the cage, beneath the animals^208^, while others used resin plates with embedded magnets placed inside the cage floor to provide mechanically stable and spatially uniform exposure conditions^63^. In contrast, other In Vivo tumor models employed cylindrical or block-shaped permanent magnets positioned externally at a controlled distance from the animal^61,202^. To avoid direct contact between the magnet and biological tissue while preserving the intended field magnitude, several In Vivo setups incorporate non-ferromagnetic spacers, most commonly titanium frames or supports ^61,63,117,148,202,209^. The use of ferromagnetic interfaces (e.g., steel plates or spacers) to homogenize the SMFs’ spatial profile effectively decreases macroscopic variability but may simultaneously create flux boundary conditions that distort natural gradient profiles, particularly at the animal cell-magnet interface. Indeed, magnetic field mapping studies demonstrate that proximity to ferromagnetic materials creates non-linear field transitions that can confound intended spatial profiles, particularly over distances comparable to cellular dimensions, leading to discrepancies between computational models, In Vitro studies, and results with animal models^210,211^. This problem is circumvented by an interposing layer of a non-ferromagnetic (relative permeability ≈ 1), which does not concentrate or shunt magnetic flux the way steel or other ferromagnets do. This way, the high-permeability boundary that would otherwise create sharp, non-linear field jumps at the interface would be eliminated. Resin fiber plates can also provide a non-magnetic rigid support to hold multiple button magnets at fixed positions and orientations, ensuring reproducibility across cages and experiments (Table 3)^63^.

Another significant problem observed In Vivo is that of field penetrability. For instance, in a trial conducted to study the influence of magnetic wraps (27 mT surface field for 48 h) on horses—results indicated, no measurable improvement in blood flow. This may be attributed to the fact that subcutaneous SMF stimulation at just 7 mm depth decayed exponentially to ≤0.05 mT—comparable to or weaker than the ambient geomagnetic field (0.025–0.065 mT)^212^. This rapid flux density decay illustrates the fundamental challenge of achieving therapeutically relevant intracellular field strengths In Vivo and the significance of the delivery method in determining the “effective” magnetic field intensity experienced by the target cells. Hence, achieving effective therapeutic field strengths at the target tissue is an uphill task, and researchers need to consider various factors like the depth of the target tissue in an average individual, alongside magnet design and placement. An effective SMF stimulation device must additionally overcome interference arising from implantable medical devices (pacemakers, insulin pumps, cochlear implants) and metallic prosthetics, which could alter the magnetic field intensity and spatial field distributions at the relevant tissue or intracellular target. Thus, incomplete and inconsistent modeling of the SMF’s spatiotemporal profile can lead to unexpected experimental outcomes, hindering the advancement of the field. This issue of “effective” magnetic field being unsatisfactory is a key reason why many In Vivo magnetic therapy experiments may yield disappointing or inconclusive results.

#### Magnetic field direction

The direction of SMF, in relation to the cell’s internal architecture and external environment, is crucial in determining the cellular response. This directional dependence arises from the intrinsic anisotropic nature of many cellular components and their orientation within tissues. Notably, reversing the magnetic field direction alters the force and torque vectors acting on all components, which significantly changes the overall dynamics of the biological system and leads to drastic alterations in the cellular response.

For instance, one finding examined cell growth in different cell lines, including five human solid tumor cell lines and two human leukemia cell lines, under the influence of SMF stimulation (Table 3)^202^. An upward-directed SMF of 200–1000 mT significantly decreased the growth of all tested human solid tumor cell lines, whereas a downward-directed SMF had no significant effect. However, leukemia cell lines, by virtue of their shape-induced anisotropy, displayed growth inhibition by both upward and downward SMF, indicating the influence of the direction of SMF stimulation on the growth rate of cells. A direction dependence was also shown in GIST-T1 mouse models, wherein upward-oriented 400–500 mT SMF stimulation for 38 days decreased tumor growth by 19.3%, but downward-oriented SMF stimulation showed no significant growth inhibition^202^.

Similarly, exposure of A549 lung cancer cells and xenograft-bearing mice to a 9400 mT SMF demonstrates a strong directional dependency of biological responses^37^. After 24 h exposure, both upward and downward 9400 mT SMFs decrease DNA synthesis in A549 cells by 14.3% and 18.6%, respectively. This can be ascribed to the Lorentz-force- based mechanism whereby strong SMF can alter the rotational velocity of DNA and its supercoil tightness, resulting in impaired DNA replication. Only the upward 9400 mT SMF is successful in increasing ROS (two-fold) and affecting G₂ arrest. Moreover, in nude mice bearing A549 xenografts, 88 h total upward 9400 mT SMF exposure (8 h/day, every other day over 21 days) decreases tumor growth by 44.7%, while downward SMF did not affect any change (Table 3). The relative orientation of SMF has ramifications not only for cellular growth rates but also modulates mitochondrial calcium concentration, membrane potential, intracellular pH, and redox balance, suggesting wider indirect effects on cellular bioenergetics and other signaling pathways^115^.

Given the significance of directionality, it is crucial to document the SMF orientation precisely. However, a major drawback of SMF-based biological studies is the omission of magnet polarity when describing the apparatus used for SMF stimulation^56,69,79,112,117,136,139^. Although schematic representations (e.g., Fig. 4B–D) illustrate magnetic field lines, they do not specify field direction due to the lack of pole identification in the sources. This may lead to major discrepancies in the results, obscuring clear interpretation and obstructing future experimentation. Even with proper and clear documentation, there is a scarcity of studies that involve the usage of SMFs in more wide-ranging directions with respect to cell culture, limiting the scope of understanding vis-à-vis directional dependency of cellular response. To wit, the electromagnetic systems depicted in Fig. 4 and Fig. 5 have limited applicability so far as the orientation of the applied magnetic field is concerned. Orienting the sample is not always possible. This requirement for a more widely applicable system is fulfilled by a more modular exposure system—Tri-axial (3-Coil) Helmholtz Configuration (Fig. 5H). An advantage of this three-axis system is its ability to generate a magnetic field of virtually any intensity and orientation in three-dimensional space. This setup allows for further exploration of the directional dependence of cellular responses upon SMF stimulation. More widespread usage of such exposure setups would make studies more informative and further efforts aimed at a therapeutic SMF treatment.

#### SMF stimulation intensity and duration

SMF intensity is directly proportional to the magnitude of force/torque experienced by the sub-cellular target. This fact is reflected in In Vivo studies as well, for example, exposure to 600 mT stimulation for 2–3 months resulted in a 50% reduction in growth, whereas 300 mT stimulation over the same period showed no substantial effect on tumor growth in transgenic PyMT mice, corroborating the significant role of SMF intensity in therapeutic outcomes (Table 3)^63^. Duration is yet another parameter that, in many instances, can make a huge biological difference. For example, human pituitary adenoma GH3 cells exhibit temporally dependent growth suppression under magnetic field exposure, requiring extended exposure periods (multiple weeks) to achieve maximal inhibitory effects, underscoring the influence of treatment duration in therapeutic applications (Table 1)^139^. In addition, In Vivo mice bearing A549 human lung tumor and GIST-T1 gastrointestinal stromal tumor, when exposed to an SMF of 9400 mT for 88 h and 200 h, respectively, in a vertically upward direction, exhibited tumor growth inhibition of 44.7% and 62.88%, respectively (Table 3)^37,213^. Furthermore, the impact of different exposure patterns was studied in mice bearing LLC-1 lung cancer. One experimental group received intermittent SMF stimulation (587 mT for 120 min on days 3, 6, and 9), achieving a 46% tumor volume reduction (19.2 ± 4.8 mm² vs. control: 35.4 ± 12.4 mm²). In contrast, daily SMF stimulation (587 mT for 35 min) showed no significant growth inhibition (31.3 ± 7.9 mm² vs. control: 35.4 ± 12.4 mm²) (Table 3)^61^. This suggests that the inhibitory effect of SMF on tumor growth is dependent on the duration of stimulation.

#### Ambient environmental conditions

Ambient environmental conditions are of the essence in all SMF-based biological studies but assume a more pivotal role in studies involving electromagnets since joule heating is a core issue in them, which can in turn perturb both CO₂ concentrations and humidity in a cell culture. Electromagnet-based setups often include extensive incubating conditions, especially for longer periods of observation, to maintain not only precise temperature but also proper gas composition (CO₂) and humidity—to prevent evaporation and pH shifts in cell culture media.

Various types of setups depicted in Fig. 5 involve different incubators for a combination of the three factors— temperature, humidity and CO₂. Recording these conditions, an ideal investigation would limit confounding variables, isolating true SMF effects and enhancing reproducibility.

#### Establishing controls

To establish a control condition, spatial decoupling of magnetic influence is essential. This is achieved through two primary methodological approaches: (1) physical isolation, where control samples are housed in separate incubators^65,76,77,90,127,140,153^ or temperature-controlled chambers^22,55,112,118,214^ to ensure complete removal from the experimental magnetic field environment, and (2) distance-based attenuation, where control cultures are positioned at predetermined distances (typically 30–40 cm) from the magnetic source within the same incubator^62,137,202,203^. Additionally, some studies construct chambers replicating the magnetic field setup—such as unmagnetized neodymium plates^117,215^ and solenoids^66,67^, Merritt-like coils^207^, or stainless-steel chambers^40,117,213^—without applying a magnetic field or power and placed inside an incubator. However, most studies reviewed in this work use the ambient geomagnetic field as the control condition, but many of them lack detailed descriptions of these control conditions. Some acknowledge the presence of the geomagnetic field in the laboratory^53,54,59,60,64,68–70,79,89,114,116,131,136,138,139,141,142,148,205^ while others refer to a “no SMF condition” without clarifying how the geomagnetic field was accounted for^56,63,88,113,147,209^. The geomagnetic field is relatively weak (being ~0.025–0.065 mT depending on geographic location) but is not entirely zero^216^. This implies that its significance would be particularly accentuated in the case of studies involving hypomagnetic fields. This is validated by a study reporting that when Human neuroblastoma (SH-SY5Y) cells are exposed to a hypomagnetic field (<500 nT), their G1-phase is accelerated, leading to a significant increase in proliferation relative to geomagnetic (≈50 µT) conditions^197^. Conversely, in HT-1080 cells, a 4-day exposure to a 0.0005 mT field (shielded) resulted in growth inhibition in comparison to control conditions (geomagnetic 0.045 mT)^115^. Additionally, a substantial body of work also indicates that intracellular signaling is altered in the presence of small magnetic fields, shielded from the geomagnetic field^217–220^. Moreover, low-intensity SMFs can induce ROS generation and form chemical products based on the radical pair mechanism^47,221^, further necessitating meticulous documentation of accounting for or nullifying the geomagnetic field to interpret experimental results effectively.

Several techniques can be used for nullifying the geomagnetic field inside the experimental volume. Magnetic shielding, wherein the experimental setup is enclosed within sheets made of high-permeability ferromagnetic materials, can nullify the geomagnetic field. These shielding materials redirect magnetic flux lines through their high-permeability structure, effectively concentrating external magnetic fields within the shielding material and preventing field penetration into the enclosed volume in which experiments can be conducted. Sheets can be formed by Mu-metals (~77% nickel, 16% iron, 5% copper, and 2% chromium or molybdenum)^222^. Nested concentric layers of Mu-metal can significantly reduce the magnetic field inside the setup, creating a nearly field-free environment in the experimental volume^59,223,224^. An alternative approach employs compensating coil systems to achieve near-zero magnetic field environments through active field cancellation. These systems utilize multi-axis coil configurations, including Helmholtz coil arrays or Merritt coils. By adjusting the current magnitude and polarity in each coil pair, these systems generate counteracting magnetic fields to cancel out the geomagnetic field^223,225^. This type of system is fundamentally similar to the exposure setup depicted in Fig. 5H. These coil systems offer flexibility over passive shielding but require stable power supplies and calibration to account for thermal drift. A straightforward way to negate the geomagnetic field is through astatisation using permanent magnets: in this modality, strategically positioned permanent magnets generate counteracting magnetic fields to neutralize Earth’s magnetic influence within the experimental volume^223^. The choice and implementation of these modalities are dictated by the scientific objectives of the study, required field characteristics, practical constraints, and available resources.

The inconsistencies observed between studies investigating the influence of SMFs on cells may partly stem from incomplete and inconsistent accounting for the geomagnetic field. For example, methods such as astatisation with permanent magnets may be more susceptible to environmental fluctuations (e.g., temperature, mechanical vibration) and may not be able to neutralize magnetic fields with the same precision as compensating coils (which can be finely adjusted and monitored, providing stable and uniform fields). Such differences can influence the reproducibility of experimental findings, which illustrates the importance of a comprehensive accounting of experimental parameters while performing meta-analyses of SMF studies.

#### The magnetic material used

The material composition of the permanent magnets used is a key factor that dictates the magnetic field intensity experienced by those cells, thereby influencing their response to SMF stimulation. Studies sometimes mention the use of a “permanent magnet” without specifying the composition and grade of the material used to compose the magnet^56,112,122^, making it difficult to precisely computationally model or experimentally replicate the spatiotemporal profile being generated. Neodymium-Iron-Boron (NdFeB)^104^ is the most widely used material in previous studies owing to its remarkable coercivity, which makes it resistant to becoming demagnetized under an external magnetic field, and high remanence, due to which it retains residual magnetization even after the external magnetic field is removed^226^. These properties make NdFeB a highly effective candidate for generating stable magnetic fields. Some studies misleadingly describe NdFeB as “neodymium”, even though the high reactivity of pure neodymium renders its standalone use in experimental settings implausible.

#### Documentation of supporting experimental apparatus

Innovative configurations have been employed across SMF-based studies to tailor the SMF stimulation as intended. But complete information on all the equipment used is a prerequisite for experimental reproducibility and data interpretation. When a custom apparatus is used for positioning or holding purposes to prevent ferromagnetic interference, or for other purposes^205^. Such an apparatus may play an important role in maintaining the spatiotemporal magnetic field profile being experienced by the cells, and hence changes in design may have ramifications on results. Hence, omission of details makes it impossible to replicate the setup, paving the way for discrepancies in experimental outcomes.

#### Conclusions

Complete recording, control, and consideration of the above-mentioned physical and physiological parameters in SMF-based studies should emphasized. The above discussion highlights that SMF stimulation has tumor-treating potential, but often incomplete reporting and inconsistent protocols have led to the incorrect notion that magnetic fields produce ‘mysterious’ effects on tumors. Incomplete documentation of various parameters, both physical and physiological, is the predominant impediment in experimental reproducibility – ultimately giving rise to discrepancies in results from studies that appear to use identical methodologies. This lack of rigor in documentation complicates accurate evaluation of SMF stimulation as a modality with potential use in tumor treatment. With many papers reporting an influence of SMF stimulation on tumor health, establishing a comprehensive database is hence important. Such a database must systematically record the responses of different cell lines and tumor models to specific SMF spatiotemporal profiles, explicit details about the treatment modality and all accompanying conditions in order to identify best course of action for in clinical settings.

## Future outlook and summary

The intracellular response to SMF stimulation relies on three biophysical principles: (1) the torque generated when the magnetic dipole moment of an intracellular species is exposed to an external magnetic field, (2) the Lorentz force which arises due to the directional movement of a charged intracellular species and (3) the radical-pair mechanism, in which the relative fraction of singlet and triplet electronic states of a (typically redox-active) biochemical species is altered by an external magnetic field, eventually causing ROS accumulation. Depending on the spatiotemporal profile of SMF used, these mechanisms can influence a wide range of cellular functions, including cell membrane integrity, [Ca²⁺] influx, cytoskeletal reorganization, ROS concentration and DNA integrity. Notably, many of the intracellular signaling pathways targeted by SMF stimulation are also targeted by well-established modalities, including reversible electroporation therapy, which permeabilizes cell membranes^227^, TTField therapy, which disrupts cytoskeletal organization^228^, and photodynamic therapy, which enhances ROS-mediated cytotoxicity^132^. As further research takes place into the intracellular targets of SMF stimulation, it is likely that mechanisms overlapping with other treatment modalities are discovered, further enhancing its attractiveness as a tumor-treating therapeutic modality. In this section, we present a compilation of open biophysical questions about the application of SMFs for tumor treatment (Table 5).

SMFs can target intracellular species with anisotropic magnetic susceptibilities, leading to the emergence of a magnetic torque. Even though the influence of SMF stimulation on ion channels through this mechanism can alter local [Ca^2+^] fluxes, its influence on the fluxes of other ions into the cell (including H^+^, Mg^2+^, K^+^, Na^+^, PO_4_^3–^, HCO_3_^–^) and the structure of ion-channels is so far unclear (Table 5, **Question 1**). This is important for high-intensity SMF stimulation, as it is expected to influence ionic diffusion through the Lorentz force. Although SMF stimulation appears to cause changes in the spatial distribution of charged species (FAK phosphorylation is altered in isolated human umbilical artery smooth muscle cells upon 5 mT SMF stimulation for 48 h^150^ and Fe (II) and Fe (III) concentrations decrease upon 10 mT SMF stimulation over 24 and 48 h in MCF-7 cell lines^114^), a deeper understanding of ion-SMF interactions will allow the emergence of a cellular-scale map highlighting how different spatiotemporal profiles of SMF stimulation lead to changes in bioelectrochemistry.

**Table 5:** Open questions regarding the influence of SMFs on tumor biophysics.

1	How do SMFs influence different ionic gradients and ion channels at the cellular scale?
2	What are the molecular intermediates that interface the radical-pair mechanism with ROS accumulation and cytoskeletal remodeling upon SMF stimulation?
3	How do intermediate filaments respond to SMF stimulation of different spatiotemporal profiles?
4	How can the Lorentz force be harnessed to influence tumor cell migration, proliferation and viability?
5	What are the mechanisms by which protein expression and small molecule signaling is altered by SMF stimulation?
6	Why do different cells respond differently to SMF stimulation?
7	What are the consequences of SMF stimulation with different 3D spatiotemporal profiles over extended periods of time on human subjects?
8	Do tumors develop resistance to SMF exposure over time, and if so, what molecular adaptations underlie this resistance?
9	What is the mechanism by which hypomagnetic fields influence the viability and proliferation of tumor cells?
10	Which intensities, durations and device designs for SMF stimulation can be used in combination with other cancer treatments such as chemotherapy, radiation therapy, photodynamic therapy and TTField therapy for synergistic tumor treatment on human subjects?

Several studies reporting decreased cancer cell viability or proliferation upon SMF stimulation also explain their results through the radical-pair mechanism, which leads to ROS accumulation, ultimately causing cytoskeletal reorganization. However, it is worth noting that the identities of molecular intermediates involved are still unknown, both in the context of which ROS species are influenced by SMF stimulation and how these species subsequently cause cytoskeletal reorganization (**Question 2**). It is also worth noting that although the influence of SMF stimulation on microtubules and actin filaments is now being researched, the response of intermediate filaments (which is also a cytoskeletal polymer) to SMF stimulation remains unclear (**Question 3**).

Of the three mechanisms discussed in Section “The molecular mechanisms of SMF action”, only a small amount of work has been performed on how Lorentz force acts on charged intracellular species, perhaps because of the large magnetic field intensities (>1000 mT) required to generate tangible differences in the trajectories of charged biomolecular species. A study on human embryonic kidney 293 T and human metastatic neuroblastoma SK-N-SH cells found that 16,000 mT SMF stimulation for 24 h decreased apoptosis through a Bax-mediated signaling pathway, initiated by an SMF-induced decrease in phase-separated condensates formed by purified Tau protein^36^. It was suggested that the Lorentz force on Tau (by virtue of its electrostatic charge and thermal velocity) was primarily responsible for inducing a decrease in phase separation. Future expansion of such findings by using high-intensity SMFs to induce Lorentz forces in other charged species, with a view to decreasing cell proliferation rates in tumors will be important (**Question 4**). Crucially, although SMF stimulation appears to also alter small-molecule^229,230^ and nucleotide-based signaling^76,136,149,208,231,232^, the molecular interfaces leading up to these changes are unclear and warrant further research (**Question 5**). Possible molecular candidates include Ca^2+^ and ROS—which can bind to small molecules and nucleotides, causing changes in protein expression and redox stress.

Irrespective of the mechanisms involved, SMF stimulation can lead to several different consequences depending on the biophysical (e.g., SMF intensity, exposure duration, temperature, spatiotemporal profile of magnetic field) and physiological (e.g., cell type, cell density) parameters involved. Thus, a standardized, well-documented framework for reporting the consequences of SMF stimulation with each of these parameters is needed to enhance the reproducibility and reliability of future studies. It is also worth noting that although several different spatiotemporal profiles have been used for SMF stimulation (often leading to different biological consequences), the same SMF spatiotemporal profile can also induce different responses depending on cell type. For example, in T cell leukemia Jurkat cell lines, 4750 mT SMF stimulation for 1 h decreased the proliferation rate and lowered cytosolic [Ca^2+^]^214^. However, when the same SMF stimulation spatiotemporal profile was used on human peripheral blood mononuclear cells, no significant change in these parameters was observed. Similarly, in cancerous gastric tissues extracted from human subjects, 100 mT SMF stimulation for 1 h decreased the concentration of the antioxidant superoxide dismutase and increased that of the peroxidation marker malondialdehyde in cancerous tissue, suggesting that SMFs induce redox stress through increased ROS accumulation^122^. However, the same SMF stimulation profile caused the opposite effect in noncancerous tissue: it increased superoxide dismutase concentration and decreased that of malondialdehyde. Even a differential response on adhesion has been observed in cancerous cells (human malignant melanoma PS1273 cell) as opposed to non-cancerous ones (human fibroblasts), with the adhesion ability of the cancer line being impaired while that of fibroblasts remaining unaffected (Table 1)^146^. The differences between cancerous and noncancerous cell types that lead to such responses on exposure to the same SMF stimulation must be explained biophysically before use in a clinical context.

The same SMF spatiotemporal profile can also cause different responses among different cancer cell types. For example, SMF stimulation using a gradient of 2090 mT/m (resulting in SMF intensity between 260 and 330 mT across the petri dish) for 72 h decreased cell proliferation and adhesion to the substrate by inducing actin filament reorganization in MCF-7 cell lines but not in HeLa cell lines (Table 1)^233^. Ideally, a cancer treatment therapy using SMF stimulation would target only tumor cells. It is thus important to understand how different tumor cells respond to SMF stimulation, and to engineer SMF stimulation in a manner such that only tumor cells are influenced (**Question 6**).

Future work emphasizing SMF stimulation over long durations of time will be important because of preliminary data showing that SMF stimulation can alter the expression patterns of membrane proteins^234^ (**Question 7**). Given the heterogeneity in SMF sensitivity of the naturally diverse cell populations within tumors, addressing the question of the existence of SMF-resistant subpopulations or the development of such subpopulations through fundamental alteration of gene/protein expression levels leading to eventual adaptation to prolonged SMF-induced stresses^114^ (**Question 8**). A substantial body of work indicates that intracellular signaling is altered in the presence of small magnetic fields, shielded from the geomagnetic field^217–220^. Systematically investigating the intracellular targets and biophysical consequences of these ‘hypomagnetic’ fields using a standardized set of spatiotemporal parameters will also be worth exploring for tumor treatment (**Question 9**).

Irrespective of its potential as a standalone therapy, SMF stimulation appears to be promising for use in combination with chemotherapy and radiation therapy (Table 2) (**Question 10**). It has been shown to upregulate the expression of cell surface receptors and enhance cell membrane permeability, thereby facilitating increased drug uptake^66–69,138^. In the presence of the drugs temozolomide, doxorubicin, fluorouracil and paclitaxel, SMF stimulation led to increased ROS production and disrupted the cytoskeleton, sensitizing cancer cells to drug-induced cytotoxicity^88,114,131^. In some instances, combined treatment induced apoptosis by promoting intracellular Ca²⁺ accumulation and triggering cell cycle arrest at the G2/M phase, ultimately amplifying the therapeutic impact of chemotherapy^79,125,141^. Present research appears ambivalent^203,205,235–237^ about synergy between SMF stimulation and X-ray induced radiation therapy, and thus, more investigation is needed to ascertain the synergistic/antagonistic role played by SMFs in radiation therapy (Table 2).

Even though several open questions remain to be answered before standalone SMF stimulation can be used for cancer treatment, careful investigation at the interface of tumor biology and magnetic field biophysics can allow the emergence of the next generation of magnetoceuticals.

## Data Availability

No datasets were generated or analyzed during the current study.
